# Effect of Treadmill Perturbation-Based Balance Training on Fall Rates in Community-Dwelling Older Adults: A Randomized Clinical Trial

**DOI:** 10.1001/jamanetworkopen.2023.8422

**Published:** 2023-04-20

**Authors:** Jens Eg Nørgaard, Stig Andersen, Jesper Ryg, Andrew James Thomas Stevenson, Jane Andreasen, Anderson Souza Oliveira, Mathias Brix Danielsen, Martin Gronbech Jorgensen

**Affiliations:** 1 Department of Geriatric Medicine, Aalborg University Hospital, Aalborg, Denmark; 2 Department of Clinical Medicine, Aalborg University, Aalborg, Denmark; 3 Department of Geriatric Medicine, Odense University Hospital, Odense, Denmark; 4 Geriatric Research Unit, Department of Clinical Research, University of Southern Denmark, Odense, Denmark; 5 Department of Health Science and Technology, Aalborg University, Aalborg, Denmark; 6 Department of Physiotherapy and Occupational Therapy, Aalborg University Hospital, Aalborg, Denmark; 7 Public Health and Epidemiology Group, Department of Health, Science and Technology, Aalborg University, Aalborg, Denmark; 8 Aalborg Health and Rehabilitation Center, Aalborg Municipality, Aalborg, Denmark; 9 Department of Materials and Production, Aalborg University, Aalborg, Denmark

## Abstract

**Importance:**

Falls are common and the leading cause of injuries among older adults, but falls may be attenuated by the promising and time-efficient intervention called perturbation-based balance training (PBT).

**Objective:**

To evaluate the effects of a 4-session treadmill PBT intervention compared with regular treadmill walking on daily-life fall rates among community-dwelling older adults.

**Design, Setting, and Participants:**

This 12-month, assessor-blinded randomized clinical trial was conducted from March 2021 through December 2022 in Aalborg University in Denmark. Participants were community-dwelling adults 65 years or older and were able to walk without a walking aid. Participants were randomized to either PBT (intervention group) or treadmill walking (control group). Data analyses were based on the intention-to-treat principle.

**Interventions:**

Participants who were randomized to the intervention group underwent four 20-minute sessions of PBT, including 40 slip, trip, or mixed slip and trip perturbations. Participants who were randomized to the control group performed four 20-minute sessions of treadmill walking at their preferred speed. The 3 initial training sessions were completed within the first week, whereas the fourth session was performed after 6 months.

**Main Outcomes and Measures:**

Primary outcome was the daily-life fall rates that were collected from fall calendars for the 12 months after the third training session. Secondary outcomes were the proportion of participants with at least 1 fall and recurrent falls, time to first fall, fall-related fractures, fall-related injuries, fall-related health care contacts, and daily-life slip and trip falls.

**Results:**

A total of 140 highly functioning, community-dwelling older adults (mean [SD] age, 72 [5] years; 79 females [56%]), 57 (41%) of whom had a fall in the past 12 months, were included in this trial. Perturbation training had no significant effect on daily-life fall rate (incidence rate ratio [IRR]: 0.78; 95% CI, 0.48-1.27) or other fall-related metrics. However, there was a significant reduction in laboratory fall rates at the posttraining assessment (IRR, 0.20; 95% CI, 0.10-0.41), 6-month follow-up (IRR, 0.47; 95% CI, 0.26-0.86), and 12-month follow-up (IRR, 0.37; 95% CI, 0.19-0.72).

**Conclusions and Relevance:**

Results of this trial showed that participants who received an 80-minute PBT intervention experienced a statistically nonsignificant 22% reduction in daily-life fall rates. There was no significant effect on other daily-life fall-related metrics; however, a statistically significant decrease in falls was found in the laboratory setting.

**Trial Registration:**

ClinicalTrials.gov Identifier: NCT04733222

## Key Points

**Question:**

Does treadmill perturbation-based balance training (PBT) reduce daily-life fall rates among community-dwelling adults 65 years or older?

**Findings:**

In this randomized clinical trial involving 140 highly functioning older adults, those who received an 4-session PBT intervention (totaling 80 minutes) experienced a statistically nonsignificant 22% reduction in daily-life fall rates over a 12-month period.

**Meaning:**

Findings of this trial suggest the need for future studies to investigate possible effects of current treadmill PBT on daily-life falls.

## Teaser

This randomized clinical trial evaluates the effect of a perturbation-based balance training treadmill intervention vs standard treadmill walking on 12-month fall rates among community-dwelling older adults.

## Introduction

Falling is the leading cause of injuries in older adults, and the associated costs of treatment and rehabilitation burden society substantially.^1,2,3,4^ Among community-dwelling older adults, most falls and fall-related injuries are caused by slips and trips.^5,6,7^ Nevertheless, some of these falls are preventable, and physical exercise is considered to be one of the most efficient preventive approaches.^6,8^ A promising task-specific exercise modality is perturbation-based balance training (PBT).^9,10^ During PBT, individuals are exposed to repeated external disturbances in a safe environment that are intended to improve reactive responses after slips and trips (an overview of the principles and mechanisms has been described elsewhere^9,10,11^). It has been shown that just a few PBT sessions can produce substantial refinements in reactive balance control after laboratory-induced perturbations.^12,13,14,15,16,17,18^ Moreover, these adaptations can be retained for up to 12 months after training cessation.^15,19,20,21^ Nonetheless, the dose for optimal retention and generalization of adaptations still needs further evaluation.^10^ Additionally, the current evidence regarding the effects of PBT on daily-life falls is inconsistent, with some studies reporting a fall-reducing effect of nearly 50%,^22,23,24^ whereas other studies demonstrate no effects.^24,25,26,27,28,29,30,31,32,33^ Most of these trials were designed either as a pilot study or as an evaluation of other outcomes, resulting in small sample sizes (<50 participants per group).^22,23,24,25,26,27,28,29,30,31,32,33^ In addition, perturbation type, dose, and intensity vary between studies, limiting the ability to make recommendations for PBT.^22,23,24,25,26,27,28,29,30,31,32,33^

The recently published world guidelines for fall prevention highlight PBT as a future research priority.^4^ Therefore, this randomized clinical trial (RCT) aimed to evaluate the effects of a 4-session treadmill PBT intervention compared with regular treadmill walking on daily-life fall rates among community-dwelling older adults.

## Methods

### Study Design, Setting, and Participants

This assessor-blinded, parallel-group (1:1 ratio) RCT was conducted between March 2021 and December 2022 at a laboratory in Aalborg University in Denmark. The trial protocol was approved by the North Denmark Region Committee on Health Research Ethics and the Danish Data Protection Agency. All participants provided written informed consent. The trial protocol (Supplement 1) and statistical analysis plan were preregistered at ClinicalTrials.gov and published elsewhere.^34^ There were no important deviations from the protocol. We followed the Consolidated Standards of Reporting Trials (CONSORT) reporting guideline.^35^

Individuals were recruited through radio and television advertisements and snowball sampling from March through November 2021. Eligible individuals were 65 years or older, community dwelling, and able to walk without a walking aid. Individuals were excluded if they (1) had any of the following self-reported conditions: orthopedic surgery within the past 12 months, osteoporosis or osteoporosis-related fractures (low-impact hip, spine, or wrist fracture), or progressive neurological disease (eg, Parkinson or multiple sclerosis); (2) had an unstable medical condition that prevented safe participation; (3) had a severe cognitive impairment (score <8 in the Short Orientation-Memory-Concentration Test); or (4) were currently participating in another fall prevention trial.

### Randomization and Interventions

**Figure 1.**
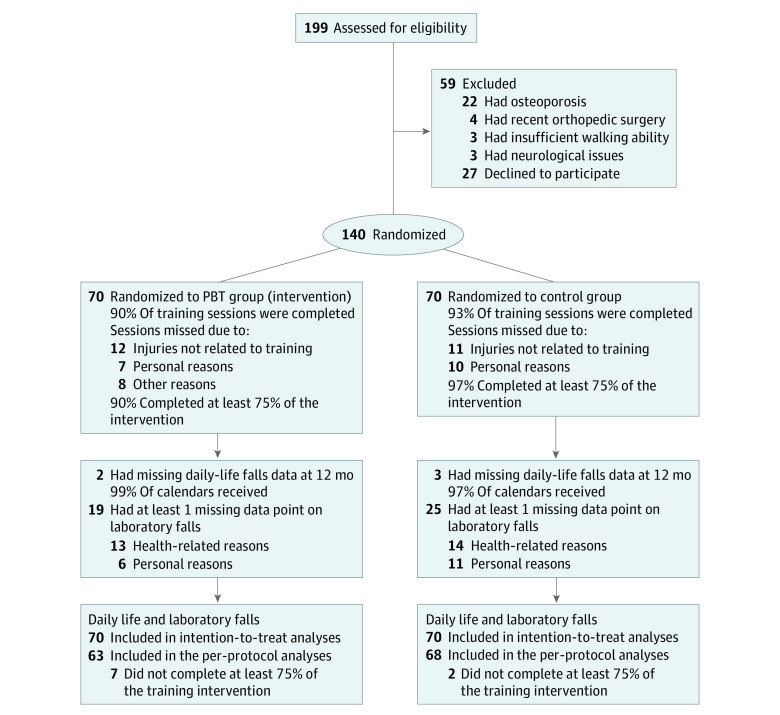
Participant Flowchart PBT indicates perturbation-based balance training.

After the pretraining assessments, participants were randomized in a 1:1 ratio to either the PBT (intervention) group or control group (Figure 1). A permuted block randomization sequence was generated in Stata (StataCorp LLC) and uploaded in REDCap, version 9.5.6 (Vanderbilt University) by a research member who was not involved in any training or testing activities. Allocation sequence concealment was ensured by a random block size of 2, 4, 6, or 8.

**Figure 2.**
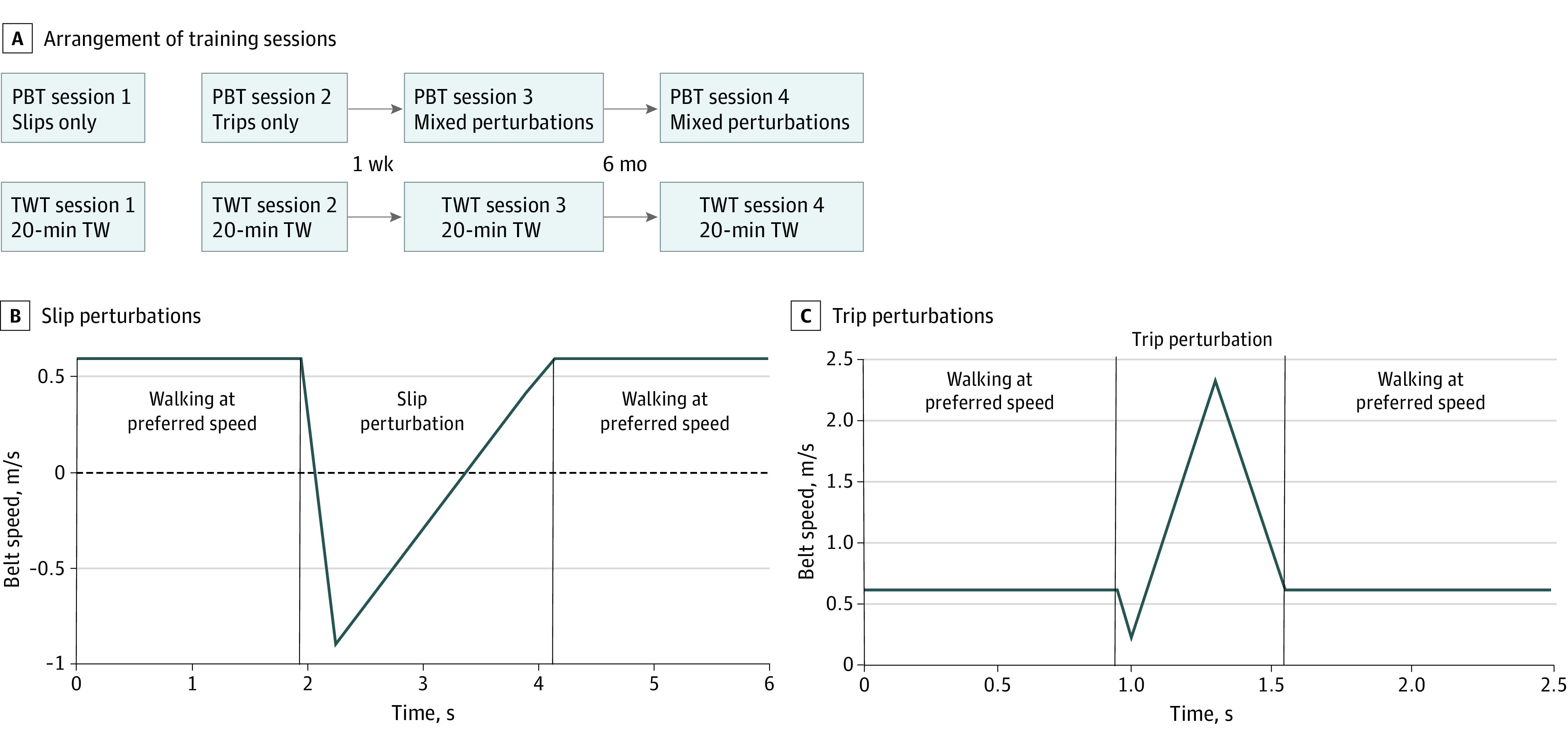
Training Schedule The figure illustrates the arrangement of the training sessions (A). During slip perturbations (B), the participant walked at their preferred walking speed when a sudden acceleration reversed the direction of the belt movement before the belt returned to the preferred walking speed. During trip perturbations (C), the participant walked at their preferred walking speed when a sudden small deceleration followed by a larger backward acceleration occurred before the belt returned to the preferred walking speed. Positive belt speed values (B and C) indicate the belt movement direction during regular walking. PBT indicates perturbation-based balance training; TW, treadmill walking; TWT, treadmill walking training.

All participants in the PBT group were assigned to four 20-minute PBT sessions, and participants in the control group were assigned to 4 treadmill walking training sessions; the initial 2 training sessions were performed on the same day immediately after the pretraining assessments. A week later, the third training session was completed, whereas the fourth was conducted after 6 months (Figure 2A). The training sessions of the intervention and control groups were conducted in the same laboratory setting. Before the first session began, participants’ preferred treadmill walking speeds were determined by increasing and decreasing the belt speed to establish the upper and lower limits for comfortable walking. The preferred walking speed was then defined as the mean of the upper and lower boundaries.^36^ eAppendix 1 in Supplement 2 provides a detailed description of the training interventions.

#### Perturbation-Based Balance Training (Intervention)

Each of the 4 PBT sessions lasted approximately 20 minutes and consisted of 40 perturbations (20 to each leg) delivered on a uniformly moving treadmill with no lateral rails (Split 70/157/ASK; Woodway). A safety harness prevented participants from falling to the ground if they could not regain balance after a perturbation. Participants experienced only slips in the first session, only trips in the second session, and randomly ordered slips and trips (mixed perturbations) in the third and fourth sessions (Figure 2A). The side (right or left leg) and timing (intervals of 10 to 50 steps between) of the perturbations were randomized to increase unpredictability. The slip perturbations were induced by a quick forward acceleration at heel strike (0% of the gait cycle), causing a reversal in the treadmill movement direction (Figure 2B). The trip perturbations were induced by a sudden deacceleration followed by a greater backward acceleration at midswing (approximately 80% of the gait cycle) (Figure 2C).

The perturbation intensity was adjusted based on participants’ preferred walking speed and was divided into 5 levels with progressively longer durations (slip) or greater accelerations (trip). The intensity was increased after every fourth perturbation if (1) the combined rating of perceived anxiety and difficulty was 4 or less, (2) the participant avoided falling during the previous 4 perturbations, and (3) the participant was comfortable increasing the intensity. However, if any criterion was not met, the intensity remained unchanged (for protocol tailoring, see eAppendix 1 in Supplement 2).

#### Treadmill Walking Training (Control)

In each of the 4 treadmill walking training sessions, the control participants performed 20 minutes of treadmill walking at their preferred walking speed. The duration of these walks matched the PBT group's time on the treadmill.

### Outcome Assessment

This RCT’s primary outcome was the daily-life fall rates that were collected from fall calendars for the 12 months after the third training session. A fall was defined as “an unexpected event in which the participant comes to rest on the ground, floor, or lower level.”^37^^(p1619)^ Participants made daily recordings in fall calendars and returned these calendars to the research team at the end of each month using preaddressed envelopes. All fall calendars were reviewed by a research assistant who was blinded to the group randomization. If a participant recorded a fall, the blinded research assistant interviewed the participant over the telephone to validate the fall and retrieve additional information about the circumstances and consequences of that fall.

Secondary outcomes included the proportion of participants experiencing at least 1 fall and recurrent falls, time to first fall, fall-related fractures, fall-related injuries, fall-related health care contacts, and daily-life slip and trip falls. A fall-related fracture was a radiologically confirmed fracture from a fall.^37^ A fall-related injury was any minor (eg, bruises, lacerations, or soreness) or major (eg, fractures or head injuries) injury from a fall. A fall-related health care contact was defined as any contact with the health care system (eg, general practitioner, emergency department, or hospital admission) associated with a fall.

At the pretraining and posttraining assessments and the 6-month and 12-month follow-up, laboratory falls were evaluated by exposing participants to level-1 slip and trip perturbations. These perturbations were recorded in slow motion (100 fps [frames per second]) with a camera (Canon M200; Canon Inc) that was positioned 2 m away, perpendicular to the center of the treadmill. The video recordings were reviewed by a blinded research assistant to determine whether the participants experienced a fall. An attempt was categorized as a fall when the participant's body posture was in a falling mode that was clearly and unambiguously stopped by the safety harness. The laboratory fall rate (number of laboratory falls per exposed perturbation) was used for analysis. The personnel responsible for the training sessions (J.E.N.) registered all adverse events related to the intervention, including any injury or unpleasantness.

### Statistical Analysis

An a priori sample size calculation was performed using a Poisson regression model in G*Power, version 3.1.9.4 (University of Kiel). To detect a significant (2-sided α = .05) difference between groups of 50% from a base fall rate of 0.85 with 80% power and an expected 20% dropout, a total of 140 participants, with 70 in each group, was needed. The expected effect size of 50% was based on previous PBT reports.^38,39,40^ The base fall rate of 0.85 was extracted from the control groups of the studies on physical exercise and fall prevention that were included in a systematic review.^8^

The statistical analyses were conducted in collaboration with an external statistician according to a preregistered statistical analysis plan using Stata, version 17.0 (StataCorp LLC). The analyses were based on the intention-to-treat principle. Two-sided *P* < .05 was considered to be significant.

Between-group differences in count outcomes were analyzed using a negative binomial regression with person-years of follow-up as an offset variable. We applied the negative binomial regression as the data fit better with this model than with the preregistered Poisson regression with bootstrapping. A sensitivity analysis using the preregistered approach was conducted. Binary daily-life fall outcomes were evaluated using Poisson regression with robust error variance.^41^ The between-group difference in time to first fall was analyzed with a Cox proportional hazards regression model (the eFigure in Supplement 2 provides the survival plot). Data for participants with no falls were censored at the end of follow-up. The proportional hazards assumption was tested statistically, and data met this assumption (χ^2^ = 1.55; *P* = .21).^42^ The Stata codes used for analyses are available in eAppendix 2 in Supplement 2.

Preregistered sensitivity analyses were conducted for each daily-life fall outcome, adjusting for age, sex, and previous falls and following the per-protocol principle, among participants who completed at least 75% of the training sessions. Sensitivity analyses that were decided after data collection included fall rates after stratifying participants based on their fall history and laboratory fall rates after multiple imputations.

## Results

### Enrollment and Adherence

Of the 199 individuals screened for eligibility, 140 were included (mean [SD] age, 72 [5] years; 79 females [56%] and 61 males [44%]) and randomized to either the PBT or control group (Figure 1). Fifty-seven participants (41%) had a fall in the past 12 months before study enrollment. The baseline characteristics of the participants were comparable between groups (Table 1).

**Table 1.** Baseline Characteristics of Participants

Characteristic	Participants, No. (%)	
	PBT group (n = 70)	Control group (n = 70)
Age, mean (SD), y	72.7 (4.7)	72.0 (4.7)
Sex		
Female	41 (59)	38 (54)
Male	29 (41)	32 (46)
Frailty, median (IQR), score^a^	2 (1-3)	2 (1-3)
Living alone	23 (33)	24 (34)
With low activity level	4 (6)	6 (9)
No. of medications used, median (IQR)	2 (0-4)	3 (0-4)
Function of daily activities, median (IQR), score^b^	2 (1-2)	2 (1-2)
Had a fall within past 12 mos	28 (40)	29 (41)
Concern with falling, median (IQR), score^c^	7 (7-8)	7 (7-8)
Cognition, median (IQR), score^d^	26 (24-28)	26 (24-28)
Physical function, median (IQR), score^e^	12 (11-12)	12 (11-12)
Habitual gait speed, mean (SD), m/s	1.3 (0.2)	1.3 (0.2)
Executive function, median (IQR), s^f^	46.4 (28.8-61.3)	39.8 (26.1-61.3)

Abbreviation: PBT, perturbation-based balance training.

^a^ Tilburg Frailty Indicator: score range of 0 to 15, with a lower score indicating less frailty.

^b^ Vulnerable Elders-13 Survey: score range of 0 to 10, with a lower score indicating less vulnerable.

^c^ Short Falls Efficacy Scale: score range of 7 to 28, with a lower score indicating less concern about falling.

^d^ Short Orientation-Memory-Concentration Test: score range of 0 to 28, with a higher score indicating better performance.

^e^ Short Physical Performance Battery: score range of 0 to 12, with a higher score indicating better performance.

^f^ Time to complete the Trail Making Test Part A was subtracted from Part B time, with less time indicating better executive function.

The PBT group completed 90% and the control group completed 93% of the assigned training sessions. At least 75% (per-protocol criterion) of the training sessions were completed by 90% of the PBT group and 97% of the control group.

At 12 months, the PBT group had returned 99% (71.5 person-years) and the control group had returned 97% (70.4 person-years) of fall calendars. Nineteen participants (27%) in the PBT group and 25 participants (36%) in the control group had missing laboratory fall data. Data were treated as missing at random because the reasons for missing data and the demographic characteristics of participants with missing data were similar between the 2 groups (eTables 1 and 2 in Supplement 2).

### Daily-Life and Laboratory Falls

**Figure 3.**
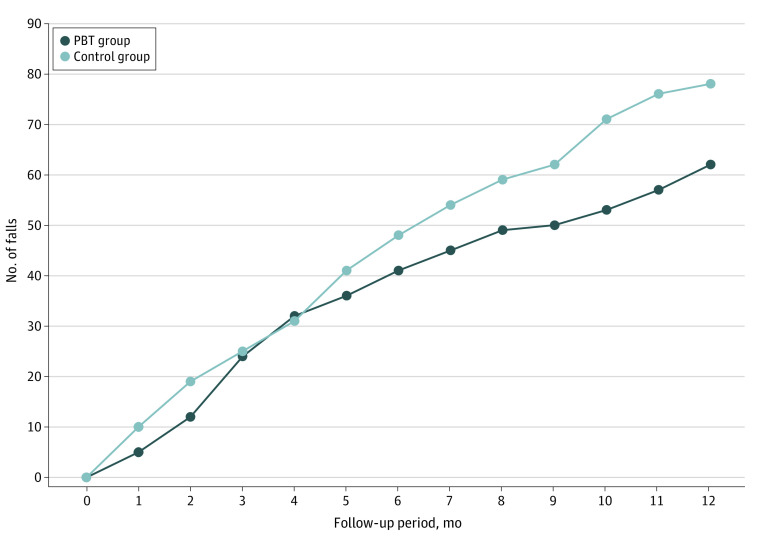
Comparison of the Number of Falls in the Intervention and Control Groups PBT indicates perturbation-based balance training.

For the primary outcome, 62 falls in the PBT group (rate of falls per person-year of follow-up: 0.90; 95% CI, 0.58-1.21) and 78 in the control group (rate of falls per person-year of follow-up: 1.14; 95% CI, 0.76-1.52) were reported, resulting in a nonsignificant 22% relative between-group difference in fall rates (incidence rate ratio [IRR], 0.78; 95% CI, 0.48-1.27) (Figure 3 and Table 2). No significant between-group differences in any secondary daily-life fall outcomes were found (Table 2).

**Table 2.** Daily-Life Fall Results From Intention-to-Treat Analysis After 12 Months

	PBT group (n = 70)	Control group (n = 70)	Absolute difference, rate (95% CI)	Relative difference (95% CI)
Person-years of follow-up	71.5	70.4	NA	NA
Falls, No. (rate [95% CI])^a^	62 (0.90 [0.58 to 1.21])	78 (1.14 [0.76 to 1.52])	−0.25 (−0.74 to 0.25)	IRR: 0.78 (0.48 to 1.27)
Participants with at least 1 fall, No. (%)	34 (49)	39 (57)	−0.07 (−0.24 to 0.09)	RR: 0.87 (0.63 to 1.20)
Participants with ≥2 falls, No. (%)	11 (16)	15 (21)	−0.06 (−0.19 to 0.07)	RR: 0.73 (0.36 to 1.48)
Time to first fall, median (IQR), d	94 (55 to 163)	123 (49 to 235)	NA	HR: 1.41 (0.87 to 2.28)
Fall-related injuries, No. (rate [95% CI])	25 (0.35 [0.19 to 0.51])	34 (0.48 [0.28 to 0.68])	−0.13 (−0.39 to 0.12)	IRR: 0.72 (0.39 to 1.34)
Participants with at least 1 fall-related injury, No. (%)	17 (24)	24 (34)	−0.10 (−0.25 to 0.05)	RR: 0.74 (0.43 to 1.25)
Fall-related health care contacts, No. (rate [95% CI])	2 (0.03 [−0.01 to 0.07])	8 (0.12 [0.03 to 0.20])	−0.09 (−0.18 to 0.00)	IRR: 0.25 (0.05 to 1.21)
Participants with at least 1 fall-related hospital contact, No. (%)	2 (3)	7 (10)	−0.07 (−0.15 to 0.01)	RR: 0.29 (0.06 to 1.33)
Participants with at least 1 fall-related fracture, No. (%)	1 (1)	2 (3)	NA^b^	NA^b^
Daily-life slip falls, No. (rate [95% CI])	9 (0.13 [0.03 to 0.23])	13 (0.19 [0.06 to 0.32])	−0.06 (−0.23 to 0.10)	IRR: 0.68 (0.25 to 1.89)
Daily-life trip falls, No. (rate [95% CI])	27 (0.39 [0.19 to 0.59])	33 (0.49 [0.25 to 0.72])	−0.09 (−0.47 to 0.28)	IRR: 0.80 (0.40 to 1.63)

Abbreviations: HR, hazard ratio; IRR, incidence rate ratio; NA, not applicable; PBT, perturbation-based balance training; RR, risk ratio.

^a^ Primary outcome. The rate is the falls per person-year of follow-up.

^b^ Not enough events to conduct analysis.

The laboratory fall rates were similar at baseline but were significantly lower in the PBT group than in the control group at the posttraining assessment (IRR, 0.20; 95% CI, 0.10-0.41), 6-month follow-up (IRR, 0.47; 95% CI, 0.26-0.86), and 12-month follow-up (IRR, 0.37; 95% CI, 0.19-0.72). All laboratory fall rate results are shown in eTable 3 in Supplement 2.

### Sensitivity Analyses

The Poisson regression with bootstrapping yielded results that were comparable to those of the negative binomial regression for all count outcomes (eAppendix 3 in Supplement 2). Moreover, both preregistered sensitivity analyses yielded no difference in results in daily-life fall outcomes and primary analyses (eTables 4 and 5 in Supplement 2). The analysis of participants with a history of falls showed similar fall rates between the PBT (n = 28) and control (n = 29) groups (IRR, 0.91; 95% CI, 0.50-1.65). However, 45% fewer falls (IRR, 0.55; 95% CI, 0.28-1.08) were registered in the PBT group (n = 42) compared with the control group (n = 41) among participants without a history of falling (eTable 6 in Supplement 2). Multiple imputations of the laboratory fall outcomes did not significantly change the estimates (eAppendix 4 in Supplement 2).

### Safety

No serious adverse events were reported in the present RCT. Nevertheless, 2 injuries occurred during PBT: a knee injury and a muscle strain in the thigh. The participant with the knee injury had 1 appointment with the general practitioner, but none of the injuries required any further health care treatment. An overview of the adverse events is available in eTable 7 in Supplement 2.

## Discussion

In this trial of a 4-session treadmill PBT intervention, there was not a statistically significant decrease in daily-life fall rates among community-dwelling older adults compared with those in the control group. There was, however, a significant reduction in laboratory fall rates associated with PBT.

The significant decrease in laboratory fall rates found in this study aligns with results of previous PBT studies.^12,13,14,15,16,17,18,43^ However, improved laboratory performance was not transferred to the daily-life falls, even though a nonsignificant 22% reduction was observed favoring the PBT group. The sample size of the current study was estimated based on an expected 50% decrease; hence, the trial was underpowered to detect a 22% between-group difference. It is noteworthy that the 22% decrease in daily-life fall rates was similar to the 23% reduction reported in a large systematic review of studies on physical exercise.^8^ In contrast to 80 minutes of total training in the PBT protocol, most studies in that systematic review applied multiple weekly sessions for 12 or more weeks.^8^ Thus, the findings of the present trial indicate that PBT is more time efficient than general exercise approaches. However, the results must be validated in larger trials with sufficient sample sizes to detect approximately 20% fall rate reductions.

Of the 4 RCTs with a primary aim of investigating the effects of PBT on daily-life falls,^23,28,32,33^ 3 used treadmill PBT and 1 used an overground walkway. These 3 RCTs found no beneficial effects on daily-life fall rates (IRR, 0.93-1.19),^28,32,33^ and 2 of these trials included only 1 type of perturbation (either slips or trips),^28,33^ diminishing the transferability of these interventions since unidirectional perturbations are not associated with improved reactive balance control after perturbations in other directions.^10,44,45^ However, 1 RCT investigated adding multidirectional treadmill PBT to usual balance training but showed no effects on daily-life fall rates (IRR, 1.11; 95% CI, 0.98-1.24).^32^ Its participants were categorized as having a high risk of falling, 70% of whom had experienced a fall in the preceding year.^32^ However, it has been shown that older adults with a history of falls may be less responsive to PBT than participants without a previous fall.^46^ In this trial, the sensitivity analysis found larger effects among participants without falls (IRR, 0.55; 95% CI, 0.28-1.08) than participants with a previous fall (IRR, 0.91; 95% CI, 0.50-1.65), suggesting that PBT may be most effective in primary prevention. However, the results join the current evidence, which has not yet demonstrated that treadmill PBT can prevent daily-life falls among community-dwelling older adults.^28,32,33^

While treadmill PBT did not show significant decreases in daily-life falls, overground PBT may be more effective.^23^ Pai et al^23^ showed that overground PBT reduced the fall rate by 49% and any falls by 51%. This finding suggested that overground walkway PBT offers superior adaptation transferability to daily life compared with treadmill PBT. A possible explanation is that overground walkway perturbations more closely resemble daily-life situations than perturbations induced by treadmill accelerations.^10^ For instance, recent evidence showed that repeated trips induced by treadmill accelerations did not improve reactive balance control in a subsequent obstacle-induced trip.^47^ In daily life, trips occur by obstructing the swinging leg, demanding elevation or lowering of the leg before a large forward step is taken to regain balance.^48^ In contrast, the response following a trip from a treadmill acceleration does not necessitate such an elevation or lowering of the leg before the compensatory step is taken, leading to biomechanical differences.^47^ These differences may have resulted in suboptimal adaptation transferability.^47,49^ Moreover, while treadmill slip PBT has been associated with better reactive balance control following a subsequent overground slip perturbation, greater improvements and retention were found after practicing on an overground walkway.^50^ Although treadmills may be less task specific, they are less space consuming, cheaper, and easier to operate than overground setups, making them more clinically feasible.^45^ Thus, developing more task-specific treadmill perturbations (eg, obstacle-induced trips) may facilitate clinically feasible and more effective PBT.

### Limitations

This trial has several limitations. First, participants were unblinded to group randomization due to the nature of training interventions. Given that the initial fall reporting was a participant-reported outcome, the lack of blinding may have contributed to fewer falls reported in the PBT group. However, daily-life falls were collected according to the recommendations of the Prevention of Falls Network Europe.^37^ Second, there was no established dose-response association between PBT and daily-life falls. Thus, the applied intensity and dose of the PBT were selected based on previous studies showing the sustained effectiveness of PBT in laboratory falls.^12,50,51,52^ Still, generalization to daily life may require higher training doses or intensity.^10^ Third, due to practical limitations, laboratory falls were evaluated by visual inspection of video recordings instead of the weight supported by the safety harness.^53^ However, the assessor was blinded to group randomization, reducing this limitation’s potential bias. Fourth, participants were assembled from convenience sampling and included healthy older adults; thus, the results do not generalize to frail individuals with a higher risk of fall-related injuries.

## Conclusions

In this RCT, participants who received an 80-minute PBT intervention experienced a statistically nonsignificant 22% reduction in daily-life fall rates. There was no significant effect on other daily-life fall-related metrics; however, a statistically significant decrease in falls was found in the laboratory setting. Further studies are warranted to corroborate this effect.

## Notes

Supplement 1.
Trial Protocol

Supplement 2. **eAppendix 1.** Description of Interventions Following TIDieR Guidelines **eFigure.** Plot of the Cox Survival Analysis **eAppendix 2.** STATA Codes Used in the Primary Analyses **eTable 1.** Reasons for Missing Laboratory Data in Each Group **eTable 2.** Between-Group Comparison of Participants With Missing Laboratory Fall Data **eTable 3.** Laboratory Fall Rate Results **eAppendix 3.** Results of Poisson’s Regression With Bootstrapping **eTable 4.** Results of Sensitivity Analysis Adjusting for Age, Sex, and Previous Falls **eTable 5.** Results of Sensitivity Analysis Following the Per-Protocol Principle **eTable 6.** Results of Sensitivity Analysis Stratifying Participants by Fall History **eAppendix 4.** Supplemental Methods and Results of Multiple Imputations **eTable 7.** Adverse Events Reported During the Study

Supplement 3.
Data Sharing Statement
