# Supplementary material for: Effect of Treadmill Perturbation-Based Balance Training on Fall Rates in Community-Dwelling Older Adults: A Randomized Clinical Trial
Source: JAMA Netw Open. 2023 Apr 20;6(4):e238422. doi: 10.1001/jamanetworkopen.2023.8422 (PMC10119738; doi:10.1001/jamanetworkopen.2023.8422)
Supplement: Supplement 1. — Trial Protocol [file jamanetwopen-e238422-s001.pdf]

Supplement 1: Protocol and Statistical Analyses Plan

This supplementary material includes 1) the trial protocol accepted by the ethical committee (translated from Danish to English), and 2) the statistical analysis plan that is available at ClinicalTrails.gov (NCT04733222).

Moreover, a trial protocol have also been published in the BMJ Open journal:

Nørgaard JE, Andersen S, Ryg J, et al. Effects of treadmill slip and trip perturbation-based balance training on falls in community-dwelling older adults (STABILITY): Study protocol for a randomised controlled trial. *BMJ Open*. 2022;12(2):1-10. doi:10.1136/bmjopen-2021-052492

Original title

The effects of perturbation-based balance training on daily life falls among community-dwelling older adults: An assessor-blinded, randomized clinical trial

Lead invesitigator

Jens Eg Nørgaard,

Jens Eg Nørgaard, Cand.Scient. Sports Science, PhD student,

Department of Geriatric Medicine, Aalborg University Hospital

Hobrovej 18-22, 9000, Aalborg

Building 6, 2nd floor

Phone: +45 31 95 18 10

Research team

Stig Andersen, Department of Geriatric Medicine, Aalborg University Hospital and Department of Clinical Medicine, Aalborg University, DK-9000 Aalborg, Denmark; ORCiD: 0000-0003-3632-5213

Jesper Ryg, Department of Geriatric Medicine, Odense University Hospital, DK-5000 Odense, Denmark; Geriatric Research Unit, Department of Clinical Research, University of Southern Denmark, DK-5000 Odense, Denmark; ORCiD: 0000-0002-8641-3062

Jane Andreasen, Department of Physiotherapy and Occupational Therapy, Aalborg University Hospital, DK-9000 Aalborg, Denmark; Public Health and Epidemiology Group, Department of Health, Science and Technology, Aalborg University, DK-9000 Aalborg, Denmark; Aalborg Health and Rehabilitation Center, Aalborg Municipality, DK-9000 Aalborg, Denmark; ORCiD: 0000-0001-8000-1553

Anderson de Souza Oliveira, Department of Materials and Production, Aalborg University, Aalborg, Denmark. ORCiD: 0000-0003-2186-8100

Andrew James Thomas Stevenson, Department of Health Science and Technology, Aalborg University, Aalborg, Denmark; ORCiD: 0000-0003-1045-4738

Mathias Brix Danielsen, Department of Geriatric Medicine, Aalborg University Hospital; Department of Clinical Medicine, Aalborg University, DK-9000 Aalborg, Denmark; ORCiD: 0000-0001-7431-5257

Martin Gronbech Jorgensen, Department of Geriatric Medicine, Aalborg University Hospital and Department of Clinical Medicine, Aalborg University, DK-9000 Aalborg, Denmark; ORCiD: 0000-0002-3189-644X

Trial location

Department of Health Science and Technology

Aalborg Univerity

Fredrik Bajers Vej 7

DK-9220, Aalborg Ø

Background

In developed countries, life expectancy and the associated age-related health challenges are increasing.[1,2] Among the age-related health challenges are falls, and approximately one-third of the older adults (≥65 years) fall at least once a year, with around 30% requiring medical attention.[3,4] Particularly problematic are the approximately 10% of falls that lead to serious injuries, such as head trauma and fractures. [3,4] Fall-related fractures often contribute to loss of functional capacity, development of fear of falling, early nursing home enrollment, reduced quality of life, and even early death.[3,6–8] Most falls and fall-related injuries among community-dwelling older adults are due to external factors related to walking (e.g., slipping on a slippery surface or tripping over a curb) [5]. Thus, interventions that effectively prevent these types of falls can be very important to the individual and society.

Considering the extensive consequences of falls, it is unsurprising that fall prevention has been a hot topic in research for decades.[9,10] Recently, perturbation-based balance training (PBT) has gained attention as a potentially effective fall preventive strategy.[11,12] For example, one study showed that a single PBT session consisting of 24 slip perturbations reduced the incidence of laboratory falls from 42.5% at the first perturbation to 0% at the last.[13] This type of "trial-and-error" training can facilitate rapid adaptations in the central nervous system so that the response to slipping or tripping is not purely reactive but is based more on proactive motor programs.[14] Furthermore, the aforementioned study showed that the ability to withstand falls was largely maintained for up to one year.[13] The same researchers have also shown that the training effect can be generalized to other situations and surfaces, such as not falling on a slippery floor (oil-contaminated vinyl floor).[15] However, most interestingly, one single PBT session has been shown to halve the fall rate in the subsequent year (IRR: 0.50, 95% CI 0.26 to 0.93) compared to a control group.[16] Currently, physical training is considered the most cost-effective approach to fall prevention.[9,10] However, it has been continuously shown that long-term adherence of older adults to exercise interventions is a problem, which inhibits the long-term effect.[17] Since PBT has an apparent "vaccine-like" effect, the effectiveness of this intervention is not dependent on self-motivated participation in comprehensive exercise interventions.[16] Furthermore, senior citizens are at increased risk of experiencing serious consequences from infections, which has been highlighted during the COVID-19 pandemic.[18] One of the primary infection prevention initiatives is social distancing, which is why the cost-effective group-based exercise intervention is currently inappropriate.[19] On the other hand, PBT is performed one-to-one, limiting the potential of getting infected during training.

However, an independent research entity has not replicated the almost-too-good-to-believe effect of PBT on the fall rate.[16] In this experiment, we will investigate the effect of PBT. Moreover, the effects of PBT on other physical, cognitive, and sociopsychological factors will also be evaluated.

Primary outcome

The *trial's primary aim* is to investigate the effect of *PBT* on the fall rate *over* 12 months among *older adults* (≥65 years), compared to a control group that walks without perturbations.

Secondary outcome

We also want to investigate other secondary fall parameters, including fall risk (***the*** number who fall one or more times), fall-related fractures (***the*** number who experience one or more fall-related fractures)**,** and fall-related hospital contacts (***the*** number who experience one or more falls-related hospital contacts). In addition, we will do physical, biomechanical, neurophysiological**,** and cognitive tests to elucidate possible mechanisms behind a potential fall reduction. Finally, we will collect social-psychological measurements to demonstrate ***how PBT*** affects other relevant parameters.

Hypotheses

We expect that PBT can reduce the fall rate by 50% in the following 12 months among community-dwelling older adults, compared to a control group that performs regular treadmill walking.[16] We also hope to show that PBT can change participants' corticospinal signals, cognitive function, fear of falling, and quality of life; however, this is only speculation as this has not yet been investigated.

Methods

Design

The study will be an assessor-blinded, randomized clinical study. We use a randomized clinical intervention study, which contributes to high-quality evidence.[34] The trial is assessor-blinded, which will reduce the risk of observer bias, improving the trial design.[34] We will use a pre- and post-test design to evaluate the effect of PBT, while falls will be continuously collected using fall calendars.

Trial procedure

A research group member (JEN) will assess whether possible participants comply with the inclusion and exclusion criteria. The suitable participants who wish to participate must appear for four days at Aalborg University. The first day starts with the participants giving written consent, performing a start-up measurement, and subsequently being randomly placed in either the training or control group. Then, the training group must perform two fall-simulating training sessions with 40 sliding perturbations and 40 stumbling perturbations, respectively, while the control group must walk in the same setup without perturbations. Day two is performed within a week of day one and consists of one training session where the training group is exposed to 20 sliding and 20 stumbling perturbations in random order. The control group must again complete a training session without perturbations. Days three and four, located six and 12 months after day one, include a follow-up test to quantify the longer-term effects of fall simulation training. After six months, participants also perform a session similar to the training on day two. An overview of the experiment's timeline can be seen in figure 1, while a list of which measurements are collected for which test sessions is shown in table 1.

A subgroup consisting of the first 30 participants from each group, who voluntarily accept the request, will undergo TMS measurements before and after the training to quantify the cortico-spinal changes.

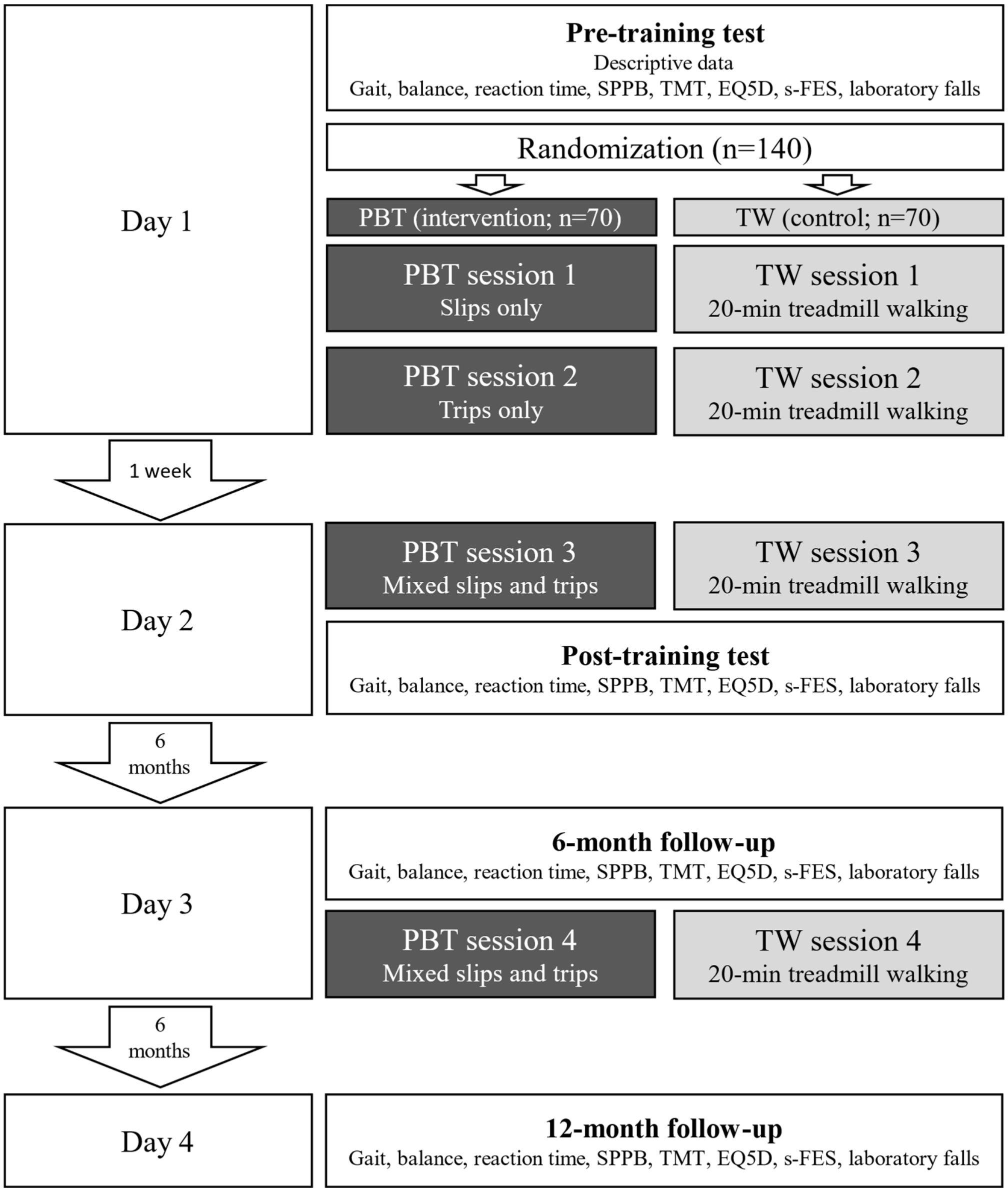

Figure 1 The study design. Dark grey boxes show the flow of the PBT group. Light grey boxed show the flow of the control group. White boxes indicates that all participants were assigned. SPPB: Short physical performance battery. TMT: Trial making test. EQ5D: EuroQoL 5-dimensions, 5-levels. s-FES: Short falls efficacy scale.

Table 1 *Assessment of outcomes across the study timeline.*

Fall assessments					
	Pre-training (T0)	Post-training (T2)	26-week Follow-up (T3)	52-week Follow-up (T4)	Continuous Assessment (T0-T4)
Falls*					X
Fall-related injuries†					X
Fall-related use of healthcare services†					X
Laboratory-induced falls†	X	X	X	X	
**Physical and cognitive assessments**					
	Pre-training	Post-training	26-week Follow-up	52-week Follow-up	Continuous assessment
Single- and dual-task gait patterns†	X	X	X	X	
Single- and dual-task balance†	X	X	X	X	
Choice stepping reaction test†	X	X	X	X	
The Short Physical Performance Battery†	X	X	X	X	
The Short Orientation-Memory-Concentration Test†	X	X	X	X	
The Trail-Making-Test Part A and B†	X	X	X	X	
Transcranial Magnetic Stimulation (substudy)†	X	X			
**Questionnaire-based assessments**					
	Pre-training	Post-training	26-week Follow-up	52-week Follow-up	Continuous assessment
EuroQoL EQ-5D-5L†	X	X	X	X	X
The Short Falls Efficacy Scale†	X	X	X	X	
The Tilburg Frailty Indexǂ	X				
Vulnerable Elders Survey-13ǂ	X				
The Physical Activity Enjoyment Scale†		X			
**Others**					
	Pre-training	Post-training	26-week Follow-up	52-week Follow-up	Continuous assessment
Anthropometric dataǂ	X				
Charlson comorbidity indexǂ	X				
Adverse events†	X	X	X	X	X
Intervention and healthcare costs (economic evaluation)†					X

* Fall rate (fall per person-year) is the primary outcome, † secondary outcome, ǂ descriptive data

Randomization

Immediately after the start-up test, the participants will be block randomized in a 1:1 ratio, in blocks of varying size (4, 6, or 8), to either the training or the control group. The randomization will be administered by a ***research team member*** who is not involved in the data collection in REDCap (Version 7.0.11).

Interventions

In this trial, the training group will undergo four fall simulation training sessions (see Figure 1). The fall-simulating training is performed on a computer-controlled treadmill. The treadmill induces perturbations by causing the backward-moving treadmill to make sudden forward (gliding perturbations) or backward (stumbling perturbations) accelerations timed using heel contacts. The sliding perturbations on the treadmill are designed to produce a forward displacement of the participant's base of support in relation to their center of gravity. The stumbling perturbations, on the other hand, are designed to shift the participants' base of support backward in relation to their center of gravity. In all training sessions, the participant will receive 40 perturbations; the first session will consist of 40 slip perturbations, the second session will consist of 40 trip perturbations, while the third and fourth sessions will consist of 40 slip and trip perturbations in random order. Before each training session begins, the participant will be informed that a perturbation might occur and that if it does, they must try to regain their balance and continue moving forward. Therefore, the participant does not know where, when or how the perturbation occurs. All participants will wear a body harness that catches them in the event of a fall, ensuring that nothing but the feet comes into contact with the ground/treadmill.

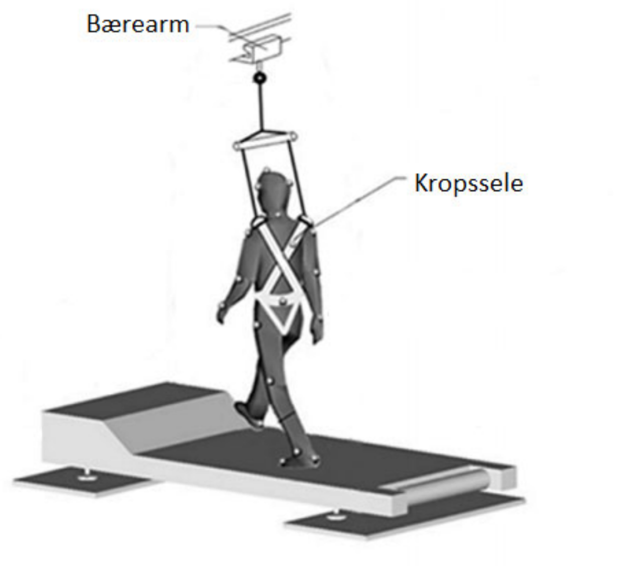

Figure 2 Illustrate the set-up of the PBT on the treadmill. The same setup was made for the control group, however they did not receive perturbations.

Participants in the control group will be instructed to walk at their preferred pace on a computerized treadmill. The control group will not be exposed to perturbations during their training.

Sample Size Calculation

A sample size calculation estimates that 70 people will be used in each group. The calculation is based on an expected fall reduction of 50% in the training group compared to the control group. Based on control groups from a large Cochrane review (≥65 years), it is assumed that the control group will have a fall rate of around 0.85. The calculation is made with an expected statistical power of 80%, a significance level of 5% and a drop-out of 20%.

Participants

Inclusion criteria

1. 65 years or more
2. Community-dwelling
3. Can walk without a walking aid

Exclusion criteria

1. Any of the following self-reported conditions: Orthopedic surgery within the past 12 months, osteoporosis or osteoporosis-related fractures (low impact hip, spine, and wrist fracture), or progressive neurological disease (e.g., Parkinson, multiple sclerosis)
2. an unstable medical condition that would prevent safe participation
3. severe cognitive impairment (a score of <8 in The Short Orientation-Memory-Concentration Test)
4. currently participating in another fall prevention trial.

The participants will be screened for the inclusion and exclusion criteria via a telephone conversation immediately before the start-up measurements are carried out. During the telephone interview, the potential participants will be asked whether they comply with the set inclusion and exclusion criteria. Exclusion criterion 3 (severe cognitive impairment) will only be screened for the pre-training test after written informed consent is provided.

Risks, adverse events, and disadvantages in the short and long term

Risk of the training interventions

Previous scientific intervention studies and systematic review articles on fall simulation training have not reported any adverse events such as falls or other injuries associated with the training [11,15,16,30,35,36]. Although perturbations may provoke falls among participants, all participants will be wearing a body harness, which ensures that nothing but the feet will be able to come into contact with the ground. In addition, all training sessions will be supervised by an experimenter who will stop the training if it is judged that a participant is too frail to complete the training.

Risk of physical measurements

The physical exercises that examine walking patterns, balance, reaction, and functional ability can pose a risk of falling. The trial leader will always support and guide the participant to minimize this risk. In addition, the experimenter will stop the testing activities if the participant is believed to be too fragile to complete the experiment.

Information from patient journals

In the experiment, no information was collected from patient records before the participant consented.

After the participants have given express written consent, information from common medicine cards and municipal benefit registers will be collected in accordance with §157, subsection of the Health Act. 13. The following information will be obtained from these registers: Age, gender, medication use, known illnesses (current and previous) and amount and nature of home care. This information will be requested via a cooperation agreement with the municipality. The information must be used to ensure an adequate description of the participants. Furthermore, the consent will only be used by personnel entitled to access in accordance with Section 157 of the Health Act.

Handling of person sensitive data

All data will be stored securely in REDCap, and permission to store data will be sought (forskninganmeldelse@rn.dk). Information about participants is stored and processed per the Data Protection Ordinance and Act to maintain the subject's physical and mental integrity and privacy, cf. Section 20 of the Committee Act.

All data will be stored securely under pseudonyms in connection with the tests. After the trial, all data will be pseudo-anonymized and stored in accordance with the Danish codex for research integrity.

Data can be shared anonymously with other researchers if it has a relevant purpose.

Recruitment and informed consent

Recruitment plan

The study is carried out in collaboration with Aalborg Municipality, from which the participants will be recruited. During the recruitment, we will give presentations about the project at the municipality's activity centres. After the presentation, those interested can sign up for a participant list, from which we will later recruit. Here, potential participants must provide their email and/or telephone number and consent to us contacting them with further information about the trial.

Recruitment will also occur through the municipality's preventive home visits, which are offered to older adults 75 years or older. The staff responsible for the home visits will provide the citizen with information about the trial, after which contact information for interested citizens will be collected. This information will be passed on to the person in charge of the trial, who will contact the interested citizens.

The recruitment will also involve: 1) handing out flyers at public institutions, leisure clubs, activity cent***er***s, medical practices***,*** and physiotherapists in and around Aalborg, 2) exposure in the press through TV, radio, sundhed.dk, forsog.dk and social media***,*** and 3) contact previous trial participants who have given consent for us to contact them in connection with other trials.

Consent

The participant will be screened for suitability via a telephone conversation, where they assess whether they comply with the established inclusion and exclusion criteria. Suppose they meet all the inclusion and exclusion criteria, except exclusion criterion 3, which will only be screened for after consent. In that case, the participant will receive written and verbal information about the trial. First, the participant will receive the written information either by email or printed on paper. A maximum of 14 days after receipt of the written information, verbal information will be given either face-to-face in a separate room without interruption or over the phone. As we recruit high-functioning older adults, a bystander is not required to overhear this, but we encourage participants to do so. Participants are given at least 24 hours after the oral or written information to consider participating in the trial. The consent will be signed in connection with the pre-training test before the test procedure is initiated. The participant can withdraw consent at any time and thus stop participating in the experiment. With the consent, the person in charge of the experiment, as well as any supervisory authority, gets direct access to obtain information in patient records, etc., including electronic records, to see information about the subject's health conditions that are part of the implementation of the research project, including self-control, quality control and monitoring which the research group is obliged to carry out, cf. section 3 of the committee act 3. The trial participant will also give separate written consent for the trial manager to collect information from the participant's FMK and municipal registers. Information from the municipal registers is obtained by requesting the municipality's elderly and disability administration to pass on personal data for use in scientific or statistical studies, cf. §10 of the Data Protection Act.

Dissemination

Before the trial is conducted, a protocol will be uploaded to www.clinicaltrials.gov. Whatever the results of the study show, we will disseminate them to a national and international interested audience through www.clinicaltrials.gov, international journals, and relevant conferences. This is because we consider a non-significant or negative result to be of the same relevance as a significant positive result.

Scientific topic

Previous scientific intervention studies with fall simulation training have not reported any harmful effects, such as falls or other injuries. However, previous studies have shown that fall simulation training can halve the fall rate in the following year. Therefore, the participants in the training group can potentially achieve these positive effects via training with minimal risk of injury and COVID-19 infection. Since our participants are characterized as well-functioning older adults, we believe it is ethically justifiable not to offer the control group a specific fall prevention intervention (walking without perturbations). This control has previously been used in a similar study 16. If it turns out that fall simulation training has a positive effect on falls, the intervention can be implemented in rural falls clinics as a vaccine-like treatment for falls. In addition to preventing falls, it may result in better old age, as the negative side effects of falls, such as reduced functionality, increased fear of falling, and decreased quality of life may also be prevented. In addition, fall-simulating training can be a cost-effective fall prevention strategy and reduce health costs. We, therefore, find the experiment ethically justifiable, and it is carried out with consideration and respect for the participants involved in the investigation.

STATISTICAL ANALYSIS PLAN (SAP) FOR:

Effects of treadmill slip and trip perturbation-based balance training on falls in community-dwelling older adults (STABILITY): a randomised controlled trial

TRIAL REGISTRATION IDENTIFIER:

NCT04733222

PROTOCOL ARTICLES PUBLISHED:

Nørgaard, J. E., Andersen, S., Ryg, J., Stevenson, A. J. T., Andreasen, J., Danielsen, M. B., ... & Jørgensen, M. G. (2022). Effects of treadmill slip and trip perturbation-based balance training on falls in community-dwelling older adults (STABILITY): study protocol for a randomised controlled trial. *BMJ Open*, *12*(2).

Version: 1.0

Date: September 30th, 2022

Study group

Jens Eg Nørgaard, MSc, Department of Geriatric Medicine, Aalborg University Hospital; Department of Clinical Medicine, Aalborg University, DK-9000 Aalborg, Denmark

Stig Andersen, Department of Geriatric Medicine, Aalborg University Hospital and Department of Clinical Medicine, Aalborg University, DK-9000 Aalborg, Denmark

Jesper Ryg, Department of Geriatric Medicine, Odense University Hospital, DK-5000 Odense, Denmark; Geriatric Research Unit, Department of Clinical Research, University of Southern Denmark, DK-5000 Odense, Denmark

Jane Andreasen, Department of Occupational Therapy and Physiotherapy, Aalborg University Hospital, DK-9000 Aalborg, Denmark; Public Health and Epidemiology Group, Department of Health, Science and Technology, Aalborg University, DK-9000 Aalborg, Denmark

Anderson de Souza Oliveira, Department of Materials and Production, Aalborg University, Aalborg, Denmark.

Andrew James Thomas Stevenson, Department of Health Science and Technology, Aalborg University, Aalborg, Denmark

Mathias Brix Danielsen, Department of Geriatric Medicine, Aalborg University Hospital; Department of Clinical Medicine, Aalborg University, DK-9000 Aalborg, Denmark

Martin Gronbech Jorgensen, Department of Geriatric Medicine, Aalborg University Hospital; Department of Clinical Medicine, Aalborg University, DK-9000 Aalborg, Denmark

Table of contents

1. Study Synopsis 13
2. Study Objectives, Hypothesis, and Outcomes 13
   2.1. Primary Objective and Outcome (if applicable) 13
   2.2. Secondary Objectives and Outcomes 13
   2.3. Descriptive Outcomes 13
   2.4. Specification of endpoints 14
      2.4.1. Primary Endpoint 14
      2.4.2. Secondary Endpoints 14
3. Study Design 14
   3.1. Sample Size 14
   3.2. Randomisation and Blinding 14
4. Study Population 14
   4.1. Subject Disposition 14
5. Data handling 14
   5.1 Missing data 15
6. Statistical Analysis 16
   Sensitivity analysis 22
      Fall outcomes 22
      Fall-related risk factor outcomes 22
   6.3. Major Protocol Deviations 22
7. Implementation of Analysis Plan 22
      STATA CODE 22
8. References .... **Error! Bookmark not defined.**

1. Study Synopsis

Falls are common among older adults and can have severe consequences such as disability, decreased quality of life, and premature death [1–4]. Perturbation-based balance training (PBT) has recently gained interest as a potential brief, effective, and sustainable fall preventive strategy [5]. During PBT, participants are exposed to repeated slips and trips during walking while wearing a safety harness in a laboratory. Two meta-analyses, looking at eight and four PBT studies, have shown a vaccination-like effect of almost 50% decreased fall rates after even small dosages (1-8 sessions) [6–8]. Nonetheless, more evidence is needed to evaluate the effects of PBT performed on a treadmill [8]. This assessor-blinded, parallel-group, randomised, controlled trial will evaluate the effects of treadmill-PBT on falls and other relevant physical, cognitive and sociopsychological factors among community-dwelling older adults.

2. Study Objectives, Hypothesis, and Outcomes

2.1. Primary Objective and Outcome (if applicable)

The primary objective of this study is to determine the effects of a four-session PBT intervention on fall rates (number of falls per person-year) in community-dwelling older adults aged 65 or older compared to treadmill walking without perturbations.

The main hypothesis is that treadmill-PBT will decrease the fall rate by up to 50% in the 12 months following the intervention compared to time-matched treadmill walking.

2.2. Secondary Objectives and Outcomes

The secondary objectives are to evaluate the effects on additional fall metrics and the potential transfer effects of PBT to other relevant physical, cognitive, and social-psychological risk factors.

The secondary fall metrics include the proportion of fallers, the time to first fall, the proportion with at least one fall-related fracture, the rate of fall-related fractures, the proportion with at least one fall-related injury, the rate of fall-related injury, the proportion with at least one fall-related healthcare contact, and the rate of fall-related healthcare contact. It is expected that the proportion of fallers is 50% lower in the PBT-group compared to the treadmill walking group.[6,9] However, we do not have enough evidence regarding the remaining fall-related outcomes to make hypothesis hereof; thus, these outcomes are considered exploratory.

The secondary fall-related risk factors included are single- and dual-task gait speed, reaction time, single- and dual-task static balance, lower extremity performance, executive function, health-related quality of life, and fear of falling. These secondary outcomes were chosen as they all have been identified as fall risk markers [10–16]. However, there is insufficient information about such outcomes following PBT; therefore, we consider these outcomes exploratory.

2.3. Descriptive Outcomes

Descriptive data include height, weight, sex, physical and cognitive function, medication usage, Tilburg Frailty Indicator, highest education level, living arrangements, and fall history, including associated injuries, everyday activity functionality (Vulnerable Elders Survey-13), physical activity levels, and home care usage. Information will be collected through a combination of self-reporting, measurements, questionnaires, and medical/municipality records. Descriptive data will be presented in a table stratified by intervention type as mean and standard deviation (normally distributed continuous variables), median and inter-quartile range (not normally distributed continuous variables), or number and percentage (categorical variables). Descriptive data will be visually compared to evaluate any potential differences between groups.

2.4. Specification of endpoints

2.4.1. Primary Endpoint

The primary endpoint will be the fall rate 12 months after completion of the third training session, and it will be collected using monthly fall calendars as recommended.[17]

2.4.2. Secondary Endpoints

Secondary outcomes and their endpoints are outlined in table 1 and 2. The secondary fall outcomes will be collected using the fall calendars for 12 months. The fall-related risk factor outcomes will be collected at the pre- and post-training test and the 26- and 52-week follow-up.

3. Study Design

This study is designed as an assessor-blinded, randomised, parallel-group (1:1 ratio), controlled trial

3.1. Sample Size

The sample size calculation was conducted in G*power (version 3.1.9.4, University of Kiel, Kiel, German) using a Poisson regression model. The calculation was made with certain assumptions (80% power, 5% significance level, 50% difference in fall rate (favouring the PBT), and 20% dropout rate) and an expected average fall rate of 0.85.[18–21] This resulted in an estimated required sample size of 70 participants in each group.

3.2. Randomisation and Blinding

After the pre-training tests, participants will be randomly allocated to either the PBT or treadmill walking group using a permuted block randomisation module in REDCap to ensure similar group sizes (Research Electronic Data Capture; version 9.5.6). Random block sizes (two, four, six, or eight) will ensure that allocation concealment is maintained. The allocation sequence will be generated by a research staff member not involved in enrolling or assigning participants to groups.

4. Study Population

4.1. Subject Disposition

One hundred forty community-dwelling older adults (70 in each group) living in and around Aalborg will be recruited via informal presentations about the trial, local and national newspapers, radio and television spots, flyer hand-outs, and snowball sampling. Participants are eligible if they are 1) ≥65 years old, 2) community-dwelling, and 3) able to walk without a walking aid. Participants will be excluded if they 1) have any of the following self-reported conditions: orthopaedic surgery within the past 12 months, osteoporosis or history of osteoporosis-related fractures (low-impact hip, spine, and wrist fracture), or progressive neurological disease (e.g., Parkinson), 2) have an unstable medical condition that would prevent safe participation, 3) have a severe cognitive impairment (a score <8 in The Short Orientation-Memory-Concentration Test)[22], or 4) are currently participating in another fall prevention trial.

5. Data handling

All data will be collected and managed using the secure, web-based software platform REDCap hosted in The Region of Northern Denmark.[23,24] The data collection forms in REDCap ensure strong data integrity by applying functions that check for mandatory information, data ranges, and alerts whenever

data violates specific limits.[24] To ensure data quality, all outcomes will be visually inspected for implausible values before the dataset is locked. Missing and out-of-range data in REDCap will also be assessed compared to original data files (paper documents for questionnaires and FysioMeter software for balance and reaction time) and corrected in cases of discrepancies. REDCap also logs every record activity, which will be used to monitor data validity.

5.1 Missing data

The number of missing observations and the associated reasons will be reported. For the primary outcome, fall rates, missing data will not be imputed; however, the analysis will be adjusted for follow-up time (days of follow-up will be used as an offset). Likewise, missing data regarding the secondary binary outcomes will neither be imputed, but the modified Poisson regression will be adjusted for person-years (days of follow-up will be used as an offset). For participants who do not return any fall calendars 0 falls and 0 person-years will be registered. However, if more than 10% of data is missing in any outcomes, a sensitivity analysis utilising multiple imputations will be conducted.

Missing data in continuous outcome are expected to be missing at random; thus, they will not be imputed as it has shown that multiple imputations do not add any benefits to a linear mixed-effects model.[25]

6. Statistical Analysis

All statistical tests will be performed using an intention-to-treat approach. Moreover, a per-protocol analysis will also be performed, including only participants who complete 75% or more of the training sessions. The secondary outcome will not be adjusted for multiple comparisons; thus, these results should be considered exploratory.

Count data will be reported as incidence rate ratios (IRR) with 95% confidence intervals (95% CI). Binary outcomes will be reported as risk ratios (RR) and 95% CIs. When appropriate, continuous variables will be reported as either mean and standard deviation or median and interquartile range.

Table 1 Variables, measures, and methods of analysis for fall outcomes.

Primary outcome					
**Variable/outcome**	**Mode of assessing**	**Time frame**	**Variable type**	**Assumption**	**Methods of analysis**
Fall rate (falls per person-year)	Fall calendars	Continuously for 12 months	Count	Equal mean and variance *	Poisson regression<br>Alternative: Bootstrapping†
Secondary fall outcomes					
**Variable/outcome**	**Mode of assessing**	**Time frame**	**Variable type**	**Assumption**	**Methods of analysis**
Proportion of fallers	Fall calendars	Continuously for 12 months	Binary	Equal mean and variance *	Poisson regression with robust error variance
Time to first fall	Fall calendars	Continuously for 12 months	Survival	Linear relationship between log hazard and covariate$^{\alpha}$	Cox proportional hazard

Fall-related fracture rate	Fall calendars	Continuously for 12 months	Count	Equal mean and variance *	Poisson regression<br><br>Alternative: Bootstrapping†
The proportion with a fall-related fracture	Fall calendars	Continuously for 12 months	Binary	Equal mean and variance *	Poisson regression with robust error variance
Fall-related injury rate (other injuries than fractures)	Fall calendars	Continuously for 12 months	Count	Equal mean and variance *	Poisson regression<br><br>Alternative: Bootstrapping†
The proportion with fall-related injuries (other injuries than fractures)	Fall calendars	Continuously for 12 months	Binary	Equal mean and variance *	Poisson regression with robust error variance
Fall-related hospital contact rate	Fall calendars	Continuously for 12 months	Count	Equal mean and variance *	Poisson regression<br><br>Alternative: Bootstrapping†
The proportion with a fall-related fracture	Fall calendars	Continuously for 12 months	Binary	Equal mean and variance *	Poisson regression with robust error variance
All-cause fracture rates	Fall calendars	Continuously for 12 months	Count	Equal mean and variance *	Poisson regression<br><br>Alternative: Bootstrapping†
The proportion with an all-cause fracture	Fall calendars	Continuously for 12 months	Binary	Equal mean and variance *	Poisson regression with robust error variance

Patient Global Impression of Change	7-item questionnaire and 11-point Likert scale; proportion "4 – somewhat better") and 0-11 points on the Likert scale (lower score indicates better performance)	52-week follow-up	7-item questionnaire: Binary<br><br>VAS:<br>Continuous<br>(Ordinal)	VAS-scale:<br>Normal distribution[ɸ]<br>Homogeneity of variance[ɸ]	7-item questionnaire: Fisher's Exact<br><br>VAS-scale:<br>Unpaired t-test<br>Alternative: Unpaired two-sample Wilcoxon test[‡]
Laboratory-induced overall fall rate	Visual inspection of video recording of a level 1 slip and trip perturbation; fall (1) or no fall (0)	Pre-training test;<br>post-training test;<br>26- week follow-up;<br>52-week follow-up	Count	Equal mean and variance *	Poisson regression<br>Alternative: Bootstrapping[†]
Laboratory-induced slip falls	Visual inspection of video recording of a level 1 slip perturbation; fall (1) or no fall (0)	Pre-training test;<br>post-training test;<br>26- week follow-up;<br>52-week follow-up	Binary	-	Fisher's Exact
Laboratory-induced trip falls	Visual inspection of video recording of a level 1 trip perturbation; fall (1) or no fall (0)	Pre-training test;<br>post-training test;<br>26- week follow-up;<br>52-week follow-up	Binary	-	Fisher's Exact

* Similarity of the calculated mean and variance; † If mean is not equal to variance; ᵅ Visual inspection of residual plots; ᶲ Examined by visual inspection of histograms and QQ-plots; ‡ If data is not normal-distributed; ᴼ Participants ID as randoms effect - only participants, who did not fall during the perturbation at pre-training test was included

Table 2 Variables, measures, and analysis methods for fall-related risk factors.

Variable/outcome	Mode of assessing	Time frame	Variable type	Assumption	Methods of analysis
Single- and dual-task gait speed	6-meter walking test; walking speed (m/s)	Pre-training test; post-training test; 26- week follow-up; 52-week follow-up	Continuous (ratio)	Normal distribution of residuals and random effects *ᶲ Homogeneity of variance⊕ᶲ	Linear mixed-effects model ᴼ
Single- and dual-task sway	30-second balance test on WBB‡; centre of pressure area (mm^2) and velocity (mm/s)	Pre-training test; post-training test; 26- week follow-up; 52-week follow-up	Continuous (ratio)	Normal distribution of residuals and random effects *ᶲ Homogeneity of variance⊕ᶲ	Linear mixed-effects model ᴼ
Choice stepping reaction time (CSRT)	CSRT on WBB‡; reaction time (ms)	Pre-training test; post-training test; 26- week follow-up; 52-week follow-up	Continuous (ratio)	Normal distribution of residuals and random effects *ᶲ Homogeneity of variance⊕ᶲ	Linear mixed-effects model ᴼ

Short physical performance battery	2x4 meter walking time, 3x10 second static balance with 3 different foot positions, and 5 chair raises; score from 0-12 (higher score indicates better performance)	Pre-training test;<br>post-training test;<br>26- week follow-up;<br>52-week follow-up	Continuous (ordinal)	Normal distribution of residuals and random effects *ɸ<br>Homogeneity of variance⊕ɸ	Linear mixed-effects model O
Trial-making-test<br>Part A and B; time and error	Part A and Part B of the Trail-making-test; time (s) and errors (n).<br>Difference in time-to-complete between Part A and Part B; time (s)	Pre-training test;<br>post-training test;<br>26- week follow-up;<br>52-week follow-up	Continuous (ratio)	Normal distribution of residuals and random effects *ɸ<br>Homogeneity of variance⊕ɸ	Linear mixed-effects model O
Short Falls Efficacy Scale	7-item questionnaire; score from 7-28 (lower score indicates better performance)	Pre-training test;<br>post-training test;<br>26- week follow-up;<br>52-week follow-up	Continuous (ordinal)	Normal distribution of residuals and random effects *ɸ<br>Homogeneity of variance⊕ɸ	Linear mixed-effects model O

EuroQoL 5D-5L	5-item questionnaire and visual analogue scale; index from 0-1 (higher score indicates better performance)<br>Visual analogue scale from 0-100 (higher score indicates better performance)	Pre-training test;<br>post-training test;<br>26- week follow-up;<br>52-week follow-up	Continuous (ordinal)	Normal distribution of residuals and random effects[*ϕ]<br>Homogeneity of variance[⊕ϕ]	Linear mixed-effects model[O]

[*] Examined by visual inspection of histograms and QQ-plots; [⊕] Examined by visually inspecting residuals plotted against fitted values; [†] If data is not normal-distributed; [ϕ] Violations of assumptions will be noted; however, no alternative method will be used, as the linear mixed-effects model is robust against such violations; [O] Participant ID as the random effect

Sensitivity analysis

Fall outcomes

For the primary outcome (fall rates) and secondary fall outcomes, a sensitivity analysis adjusting for known confounders (age, sex, and previous falls) will be conducted to evaluate the robustness of the results. Furthermore, if the count variable data is over-dispersed, a poisons regression with bootstrapping will be performed; however, a sensitivity analysis without bootstrapping will also be carried out. These analyses were planned before the commencement of data collection.

Additional fall rate sensitivity analyses 1) only including participants with no prior history of falls 12 months before study commencement and 2) only including participants with a history of falls 12 months before study commencement will also be conducted. These sensitivity analyses were planned after data collection began.

Fall-related risk factor outcomes

Before data collection commenced, it was determined to conduct a sensitivity analysis on the secondary fall-related risk factors adjusting for age, sex, and previous falls will be carried out.

6.3. Major Protocol Deviations

Major protocol deviations will be reported in the trial registration at ClinicalTrials.gov (NCT04733222), the local ethics committee, and the SAP.

7. Implementation of Analysis Plan

The data will be exported from REDCap to the statistical program STATA. An external statistician not involved in the study will assist with the statistical test.

STATA CODE

Poisson regression (example of code for fall rate):

```
poisson fallrate ib1.group, irr exposure(personyear)
```

Poisson regression with bootstrapping (example of code for fall rate):

```
poisson fallrate ib1.group, irr exposure(personyear) vce(bootstrap, reps(1000))
```

Poisson regression adjusting for age, sex, and fall history (*example of code for fall rate)*:

```
poisson fallrate ib1.group age i.sex i.prev_faller, irr exposure(personyear)
```

Poisson regression with robust error variance (*example of code for proportion of fallers)*:

```
glm faller ib1.group, fam(poisson) link(log) vce(robust) eform
```

Poisson regression with robust error variance adjusting for age, sex, and fall history (*example of code for proportion of fallers)*:

```
glm faller ib1.group age i.sex i.prev_faller, fam(poisson) link(log) vce(robust) eform
```

Cox survival analysis (example of code for time to first fall):

```
stset firstfall, failure(faller==1)
stcox group
```

Cox survival analysis adjusted for age, sex, and fall history (example of code for time to first fall):

```
stset firstfall, failure(faller==1)
stcox group age i.sex i.prev_faller
```

unpaired t-test (example of code for Global Patient Impression of Change (gpic)):

```
ttest gpic, by(group) unpaired
```

Unpaired two-sample Wilcoxon test (example of code for Global Patient Impression of Change):

```
ranksum gpic, by(group)
```

Fisher's exact (example of code for the proportion of fallers following slip perturbation at pre-training (t1)):

```
tabulate lab_slip_t1 group, exact
```

Linear mixed-effects model (*example of code for single-task gait speed)*:

```
mixed gaitspeed_st group time || record_id:, var reml
```

In case of significant interaction effect, a posthoc analysis adjusted using the Bonferroni method is employed using the following code:

```
contrast rb1.time#group, mcompare(bonferroni)
```

Linear mixed-effects model adjusting for age, sex, and fall history (*example of code for single-task gait speed)*:

```
mixed gaitspeed_st age i.sex i.prev_faller group##time || record_id:, var reml
```

In case of a significant interaction effect, posthoc tests was conducted using the following code:

```
contrast rb1.time#group
```

References

1. Christensen K, Doblhammer G, Rau R, Vaupel JW. Ageing populations: the challenges ahead. *Lancet*. 2009;374(9696):1196-1208. doi:10.1016/S0140-6736(09)61460-4

2. Salomon JA, Wang H, Freeman MK, et al. Healthy life expectancy for 187 countries, 1990-2010: A systematic analysis for the Global Burden Disease Study 2010. *Lancet*. 2012;380(9859):2144-2162. doi:10.1016/S0140-6736(12)61690-0

3. Masud T, Morris R. Epidemiology of falls. *Age Ageing*. 2001;30(54):3-7.

4. Peel NM. Epidemiology of falls in older age. *Can J Aging*. 2011;30(1):7-19. doi:10.1017/S071498081000070X

5. Luukinen H, Herala M, Koski K, Honkanen R, Laippala P, Kivelä SL. Fracture risk associated with a fall according to type of fall among the elderly. *Osteoporos Int*. 2000;11(7):631-634. doi:10.1007/s001980070086

6. Vellas BJ, Wayne SJ, Romero LJ, Baumgartner RN, Garry PJ. Fear of falling and restriction of mobility in elderly fallers. *Age Ageing*. 1997;26(3):189-193. doi:10.1093/ageing/26.3.189

7. Cumming RG, Salkeld G, Thomas M, Szonyi G. Nursing Home Admission. *Nursing (Lond)*. 2000;55(5):299-305. doi:10.1093/gerona/55.5.M299

8. Burns E, Kakara R. Deaths from Falls Among Persons Aged ≥65 Years — United States, 2007–2016. *MMWR Morb Mortal Wkly Rep*. 2018;67(18):509-514. doi:10.15585/mmwr.mm6718a1

9. Sherrington C, Fairhall NJ, Wallbank GK, et al. Exercise for preventing falls in older people living in the community. *Cochrane Database Syst Rev*. 2019;2019(1). doi:10.1002/14651858.CD012424.pub2

10. Gillespie L, Robertson M, Gillespie W, et al. Interventions for preventing falls in older people living in the community (Review). *Cochrane Database Syst Rev*. 2012;9.

11. Okubo Y, Schoene D, Lord SR. Step training improves reaction time, gait and balance and reduces falls in older people: A systematic review and meta-analysis. *Br J Sports Med*. 2017;51(7):586-593. doi:10.1136/bjsports-2015-095452

12. Lord SR, Close JCT. New horizons in falls prevention. *Age Ageing*. 2018;47(4):492-498. doi:10.1093/ageing/afy059

13. Pai Y-C, Yang F, Bhatt T, Wang E. Learning from laboratory-induced falling: long-term motor retention among older adults. *Age (Omaha)*. 2014;36(3):9640. doi:10.1007/s11357-014-9640-5

14. Tanvi B, Feng Y, Yi-Chung P. Learning to resist gait-slip falls: Long-term retention in community-dwelling older adults. *Arch Phys Med Rehabil*. 2012;93(4):557-564. doi:10.1016/j.apmr.2011.10.027

15. Bhatt T, Pai YC. Generalization of gait adaptation for fall prevention: From moveable platform to slippery floor. *J Neurophysiol*. 2009;101(2):948-957. doi:10.1152/jn.91004.2008

16. Pai YC, Bhatt T, Yang F, Wang E. Perturbation training can reduce community-dwelling older adults' annual fall risk: A randomized controlled trial. *Journals Gerontol - Ser A Biol Sci Med Sci*. 2014;69(12):1586-1594. doi:10.1093/gerona/glu087

17. Rivera-Torres S, Fahey TD, Rivera MA. Adherence to Exercise Programs in Older Adults: Informative Report. *Gerontol Geriatr Med*. 2019;5:233372141882360. doi:10.1177/2333721418823604

18. Shahid Z, Kalayanamitra R, McClafferty B, et al. COVID-19 and Older Adults: What We Know. *J Am Geriatr Soc*. 2020;68(5):926-929. doi:10.1111/jgs.16472

19. Morley JE, Vellas B. COVID-19 and Older Adult. *J Nutr Heal Aging*. 2020;24(4):364-365. doi:10.1007/s12603-020-1349-9

20. Hausdorff JM. Gait variability : methods , modeling and meaning Example of Increased Stride Time Variability in Elderly Fallers Quantification of Stride-to-Stride Fluctuations. 2005;9:1-9. doi:10.1186/1743-Received

21. Segev-Jacubovski O, Herman T, Yogev-Seligmann G, Mirelman A, Giladi N, Hausdorff JM. The interplay between gait, falls and cognition: Can cognitive therapy reduce fall risk? *Expert Rev Neurother*. 2011;11(7):1057-1075. doi:10.1586/ern.11.69

22. Snijders AH, Verstappen CC, Munneke M, Bloem BR. Assessing the interplay between cognition and gait in the clinical setting. *J Neural Transm*. 2007;114(10):1315-1321. doi:10.1007/s00702-007-0781-x

23. Yogev-Seligmann G, Hausdorff JM, Giladi N. The role of executive function and attention in gait. *Mov Disord*. 2008;23(3):329-342. doi:10.1002/mds.21720

24. Lord SR, Fitzpatrick RC. Choice stepping reaction time: A composite measure of falls risk in older people. *Journals Gerontol - Ser A Biol Sci Med Sci*. 2001;56(10):627-632. doi:10.1093/gerona/56.10.M627

25. Zijlstra A, Ufkes T, Skelton DA, Lundin-Olsson L, Zijlstra W. Do dual tasks have an added value over single tasks for balance assessment in fall prevention programs? A mini-review. *Gerontology*. 2008;54(1):40-49. doi:10.1159/000117808

26. Thapa PB, Gideon P, Brockman KG, Fought RL, Ray WA. Clinical and biomechanical measures of balance as fall predictors in ambulatory nursing home residents. *Journals Gerontol - Ser A Biol Sci Med Sci*. 1996;51(5):239-246. doi:10.1093/gerona/51A.5.M239

27. Johansson J, Nordström A, Gustafson Y, Westling G, Nordström P. Increased postural sway during quiet stance as a risk factor for prospective falls in community-dwelling elderly individuals. *Age Ageing*. 2017;46(6):964-970. doi:10.1093/ageing/afx083

28. Guralnik JM, Simonsick EM, Ferrucci L, et al. A short physical performance battery assessing lower extremity function: Association with self-reported disability and prediction of mortality and nursing home admission. *Journals Gerontol*. 1994;49(2):M85-M94. doi:10.1093/geronj/49.2.M85

29. Pai Y-C, Bhatt TS. Repeated-Slip Training: An Emerging Paradigm for Prevention of Slip-Related Falls Among Older Adults. *Phys Ther*. 2007;87(11):1478-1491. doi:10.2522/ptj.20060326

30. Liu X, Bhatt T, Wang Y, Wang S, Lee A, Pai Y-C. The retention of fall-resisting behavior derived from treadmill slip-perturbation training in community-dwelling older adults. *GeroScience*. 2021;43(2):913-926. doi:10.1007/s11357-020-00270-5

31. Groppa S, Oliviero A, Eisen A, et al. A practical guide to diagnostic transcranial magnetic stimulation: Report of an IFCN committee. *Clin Neurophysiol*. 2012;123(5):858-882. doi:10.1016/j.clinph.2012.01.010

32. Lamb SE, Jørstad-Stein EC, Hauer K, Becker C. Development of a common outcome data set for fall injury prevention trials: The Prevention of Falls Network Europe consensus. *J Am Geriatr Soc*. 2005;53(9):1618-1622. doi:10.1111/j.1532-5415.2005.53455.x

33. Gobbens RJ, MGA Schols J, ALM van Assen M. Exploring the efficiency of the Tilburg Frailty Indicator: a review. *Clin Interv Aging*. Published online 2017:12-1739. doi:10.2147/CIA.S130686

34. Schulz KF, Altman DG, Moher D. Correspondence CONSORT 2010 Statement: updated guidelines for reporting parallel group randomised trials. *BMC Med*. 2010;8(18). doi:10.1093/nq/s1-XI.274.64-d

35. Lee A, Bhatt T, Liu X, Wang Y, Wang S, Pai Y-C (Clive). Can Treadmill Slip-Perturbation Training Reduce Longer-Term Fall Risk Upon Overground Slip Exposure? *J Appl Biomech*. Published online 2020:1-9. doi:10.1123/jab.2019-0211

36. Gerards MHG, McCrum C, Mansfield A, Meijer K. Perturbation-based balance training for falls reduction among older adults: Current evidence and implications for clinical practice. *Geriatr Gerontol Int*. 2017;17(12):2294-2303. doi:10.1111/ggi.13082

37. Wasserman E. Handbook of electroencephalography and clinical neurophysiology - Risk and safety of repetitive transcranial magnetic stimulation: report and suggested guidelines from the International

Workshop on the Safety of Repetitive Transcranial Magnetic Stimulatio. 1998;108(1):1-16. doi:10.1016/0306-4522(79)90146-5

38. Rossini PM, Burke D, Chen R, et al. Non-invasive electrical and magnetic stimulation of the brain, spinal cord, roots and peripheral nerves: Basic principles and procedures for routine clinical and research application: An updated report from an I.F.C.N. Committee. *Clin Neurophysiol*. 2015;126(6):1071-1107. doi:10.1016/j.clinph.2015.02.001

39. Rossi S, Hallett M, Rossini PM, Pascual-Leone A. Safety, ethical considerations, and application guidelines for the use of transcranial magnetic stimulation in clinical practice and research. *Clin Neurophysiol*. 2009;120(12):2008-2039. doi:10.1016/j.clinph.2009.08.016

40. Counter SA, Borg E, Lofqvist L, Brismar T. Hearing loss from the acoustic artifact of the coil used in extracranial magnetic stimulation. *Neurology*. 1990;40(8):1159 LP - 1159. doi:10.1212/WNL.40.8.1159

41. Hermens HJ, Freriks B, Disselhorst-Klug C, Rau G. Development of recommendations for SEMG sensors and sensor placement procedures. *J Electromyogr Kinesiol*. 2000;10(5):361-374. doi:10.1016/S1050-6411(00)00027-4
