# Supplementary material for: Effect of Treadmill Perturbation-Based Balance Training on Fall Rates in Community-Dwelling Older Adults: A Randomized Clinical Trial
Source: JAMA Netw Open. 2023 Apr 20;6(4):e238422. doi: 10.1001/jamanetworkopen.2023.8422 (PMC10119738; doi:10.1001/jamanetworkopen.2023.8422)
Supplement: Supplement 2. — eAppendix 1. Description of Interventions Following TIDieR Guidelines eFigure. Plot of the Cox Survival Analysis eAppendix 2. STATA Codes Used in the Primary Analyses eTable 1. Reasons for Missing Laboratory Data in Each Group eTable 2. Between-Group Comparison of Participants With Missing Laboratory Fall Data eTable 3. Laboratory Fall Rate Results eAppendix 3. Results of Poisson’s Regression With Bootstrapping eTable 4. Results of Sensitivity Analysis Adjusting for Age, Sex, and Previous Falls eTable 5. Results of Sensitivity Analysis Following the Per-Protocol Principle eTable 6. Results of Sensitivity Analysis Stratifying Participants by Fall History eAppendix 4. Supplemental Methods and Results of Multiple Imputations eTable 7. Adverse Events Reported During the Study [file jamanetwopen-e238422-s002.pdf]

Supplemental Online Content

Nørgaard JE, Andersen S, Ryg J, et al. Effect of treadmill perturbation-based balance training on fall rates in community-dwelling older adults: a randomized clinical trial. *JAMA Netw Open*. 2023;6(4):e238422. doi:10.1001/jamanetworkopen.2023.8422

eAppendix 1. Description of Interventions Following TIDieR Guidelines

eReferences.

eFigure. Plot of the Cox Survival Analysis

eAppendix 2. STATA Codes Used in the Primary Analyses

eTable 1. Reasons for Missing Laboratory Data in Each Group

eTable 2. Between-Group Comparison of Participants With Missing Laboratory Fall Data

eTable 3. Laboratory Fall Rate Results

eAppendix 3. Results of Poisson's Regression With Bootstrapping

eTable 4. Results of Sensitivity Analysis Adjusting for Age, Sex, and Previous Falls

eTable 5. Results of Sensitivity Analysis Following the Per-Protocol Principle

eTable 6. Results of Sensitivity Analysis Stratifying Participants by Fall History

eAppendix 4. Supplemental Methods and Results of Multiple Imputations

eTable 7. Adverse Events Reported During the Study

This supplemental material has been provided by the authors to give readers additional information about their work.

© 2023 Nørgaard JE et al. *JAMA Network Open.*

eAppendix 1. Description of Intervention Following Tidier Guidelines

Brief name

Treadmill perturbation-based balance training (Treadmill-PBT; intervention) and treadmill walking (active control group)

Why?

Perturbation-based balance training (PBT) is a novel training approach aiming to improve the ability of older adults to make appropriate reactive gait adjustments to unexpected perturbations.[1–4] Among older adults, slips and trips are the most frequent cause of falling; thus, training interventions targeting such events are considered highly task-specific.[2,4–6] It is well documented that even small dosages of PBT lead to improvements in reactive balance control following a laboratory perturbation that can be retained for up to a year.[7–14] However, the extent to which these adaptations generalize to daily-life falls is vastly unknown. The current literature mostly consists of studies with small sample sizes, and only a few have been designed and powered to evaluate the effects on daily life falls.[15–17] Thus, further evidence regarding the effects of PBT on falls among community-dwelling older adults is warranted.[6]

What, when, and how much?

Both groups were assigned to four training sessions; the first two were conducted on the initial day, the third a week later, and the fourth after 6 months (see Figure SM2.1). The training session after 6 months was employed as a booster session, which has been shown to impede decays in training adaptations.[18] Before the initial training session, the preferred walking speed of the participant was determined by increasing and decreasing the belt speed to establish the upper and lower limit for comfortable walking. The preferred walking speed was then defined as the average of this upper and lower boundary.[19]

Treadmill perturbation-based balance training (intervention)

The PBT was delivered on a computer-controlled treadmill (Split 70/157/ASK; Woodway, Weil am Rhein, Germany). Each PBT session consisted of 40 unexpected slip and/or trip perturbations with an equal number of perturbations to each leg while walking at their preferred speed. This dose was selected as 40 perturbations has

© 2023 Nørgaard JE et al. *JAMA Network Open.*

been shown to induce large refinements in reactive balance control that were retained for 6 months.[21,22] The initial PBT session consisted of only slips and the second only trips, while the third and the fourth consisted of randomly mixed slips and trips (see Figure SM2.1). The type (slip or trip in sessions 3 and 4), timing (between 10-50 steps), and side (left or right) of perturbation was randomized to introduce contextual interference, possibly improving generalization and retention of adaptations.[23,24] The slip perturbation (backward loss of balance) was induced by a backward acceleration at heel strike (0% of the gait cycle), leading to a reversal in the movement direction of the treadmill's belt (see Figure SM2.2).[25] The trip perturbation (forward loss of balance) was triggered during the mid-swing phase (~80% of the gait cycle) by an initial deceleration followed by a larger forward acceleration (see Figure SM2.3).[26] Figure SM2.4 illustrates the timing of the slip and trip perturbations during the gait cycle. The perturbations were triggered by a contact switch placed under the left sole of the participants' heel using the Mr. Kick software (Mr. Kick III, Version 3.0, Aalborg, Denmark).

The protocol was separated into 11 blocks of two to four perturbations and was arranged in three phases: 1) the ascending phase, 2) the mixed phase, and 3) the cooldown phase (see Figure SM2.5).[25,27] This protocol was designed to facilitate overlearning and add contextual interference, which may improve the training effects.[23,25,27] The perturbation intensity was determined based on the preferred walking speed and was divided into five levels with progressively longer perturbation duration (slips) or increasing acceleration (trips) (see Table SM2.1). However, the intensity was only increased if it was tolerated by the participants (see section "Tailoring" for a detailed description).

Treadmill walking (control)

The participants allocated to the control group walked on the treadmill at their preferred walking speed for 20 minutes. The training duration of the control treadmill walking group was matched to the PBT group to ensure that reactive response alterations were not only due to enhances familiarisation with treadmill walking.

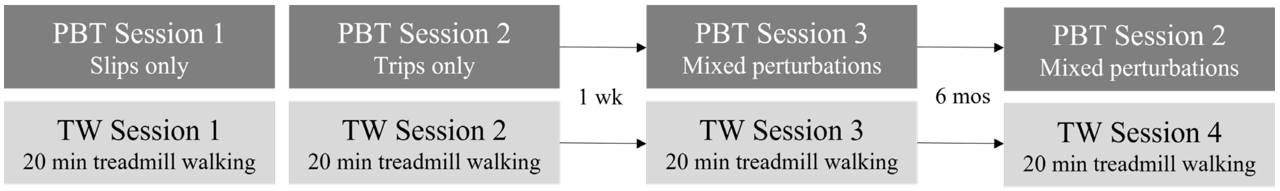

eAppendix 1, Figure 1 – The arrangement of the four training sessions in the PBT (dark grey boxes) and the control (light grey boxes) groups. PBT = perturbation-based balance training; TW = treadmill walking

© 2023 Nørgaard JE et al. *JAMA Network Open.*

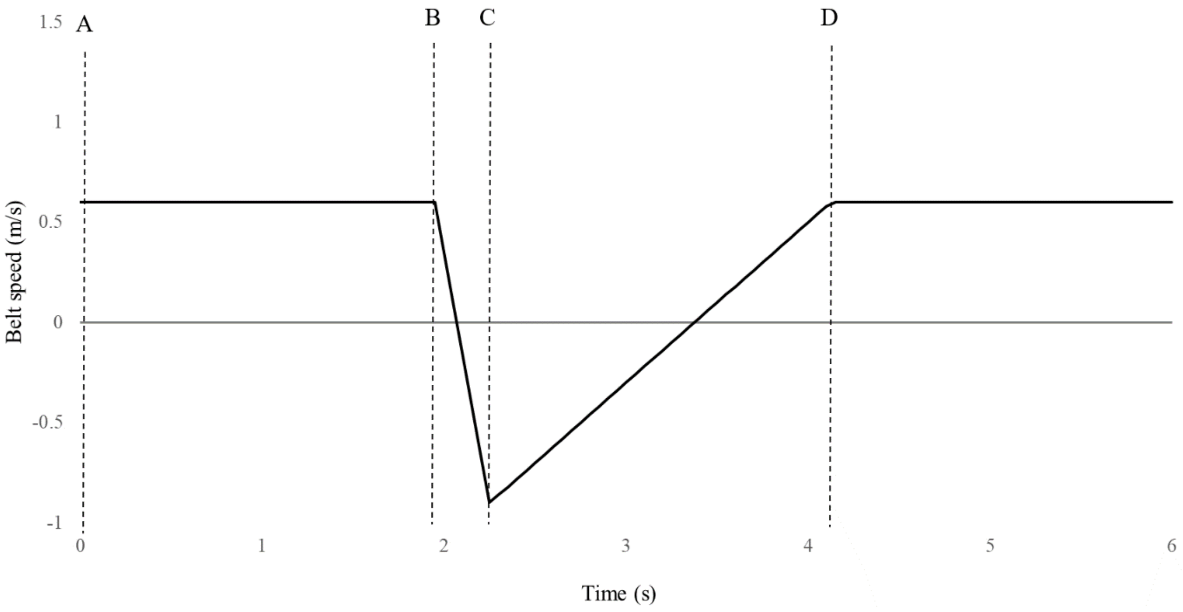

eAppendix 1, Figure 2– Illustration of the belt speed during a level 3 slip perturbation for participants with a preferred walking speed of 0.6 m/s. A-B = steady state walking. B-C = backward acceleration (deceleration of -5 m/s^2 for 0.3 seconds). C-D = forward acceleration back to the steady state walking speed (acceleration of 0.8 m/s^2 for 2.3 seconds).Figure copied from Nørgaard et al. 2022.[20]

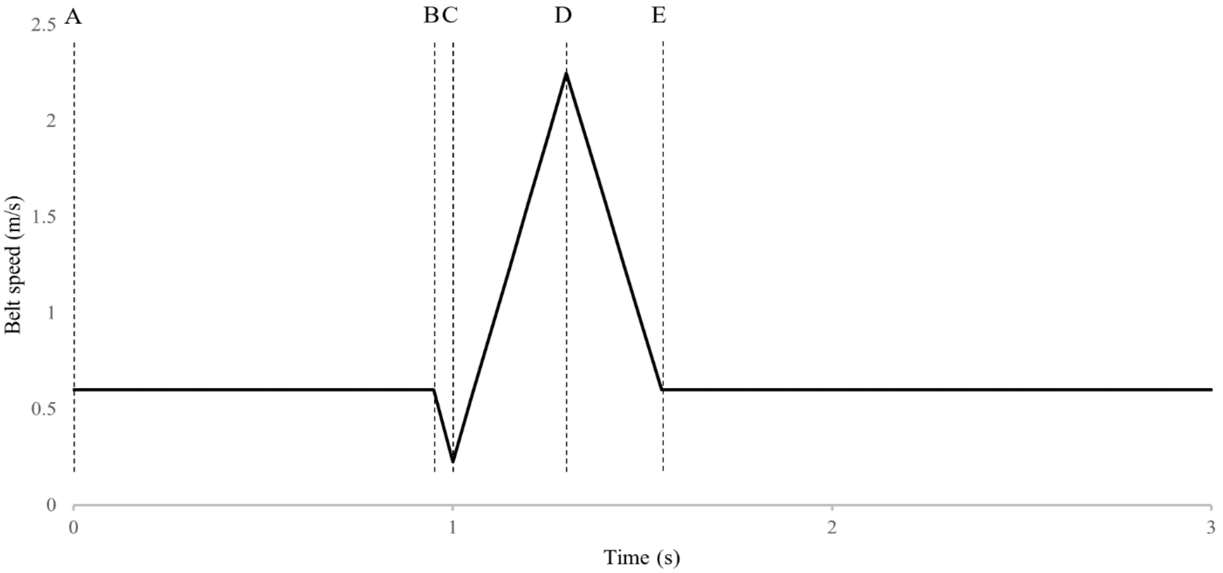

eAppendix 1, Figure 3 – Illustration of the belt speed during a level 3 trip perturbation for participants with a preferred walking speed of 0.6 m/s. A-B = steady state walking. B-C = initial deceleration (-7.5 m/s^2 for 0.05 seconds). C-D = forward acceleration (acceleration of 7.5 m/s^2 for 0.27 seconds). D-E = deceleration back to the steady state walking speed (7.5 m/s^2 for 0.22 seconds). Figure copied from Nørgaard et al. 2022.[20]

© 2023 Nørgaard JE et al. *JAMA Network Open.*

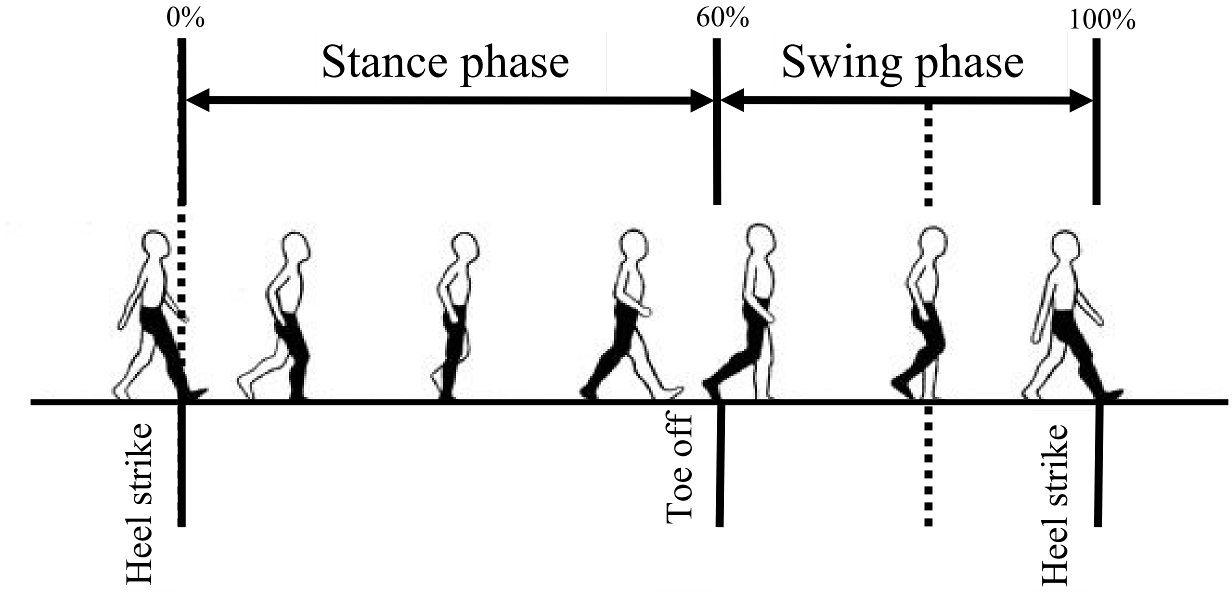

eAppendix 1, Figure 4 – Illustration of the timing of the slip and trip perturbations within the gait cycle. The first dotted line shows the timing of the slip perturbation (heel strike; 0% of the gait cycle). The second dotted line illustrations the timing of the trip perturbation (mid-swing; ~80% of the gait cycle). Figure copied from Nørgaard et al. 2022.[20]

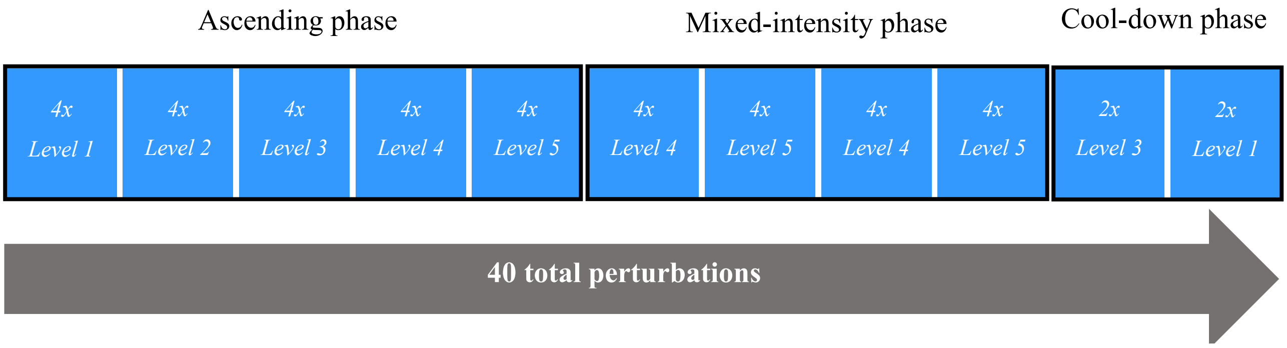

eAppendix 1, Figure 6 – The sequential arrangement of perturbation intensity levels of the PBT protocol: 1) an ascending phase in which the intensity of the perturbations progressively increases, 2) a mixed-intensity phase where the perturbation intensity varies, and 3) a cool-down phase at which the perturbation intensity decreases. However, the intensity was only increased if the participant tolerated higher levels (see for detailed description in section "tailoring)". Figure copied from Nørgaard et al. 2022.[20]

eAppendix 1, Table 1 – The perturbation profile of the different intensity levels for the slip protocol (A) and the trip protocol (B)

**A. Slip perturbation**						
Walking speed	Belt deceleration	Level 1 slip duration	Level 2 slip duration	Level 3 slip duration	Level 4 slip duration	Level 5 slip duration
≥1.2 m/s	-6 m/s^2	0.35 s	0.40 s	0.45 s	0.50 s	0.55 s
1.0 to <1.2 m/s	-6 m/s^2	0.30 s	0.35 s	0.40 s	0.45 s	0.50 s
0.8 to <1.0 m/s	-5 m/s^2	0.25 s	0.30 s	0.35 s	0.40 s	0.45 s
<0.8 m/s	-5 m/s^2	0.20 s	0.25 s	0.30 s	0.35 s	0.40 s
**B. Trip perturbation**						

© 2023 Nørgaard JE et al. *JAMA Network Open.*

Walking speed	Level 1 trip acceleration	Level 2 trip acceleration	Level 3 trip acceleration	Level 4 trip acceleration	Level 5 trip acceleration
≥1.2 m/s	7 m/s^2	8 m/s^2	9 m/s^2	10 m/s^2	11 m/s^2
1.0 to <1.2 m/s	6 m/s^2	7 m/s^2	8 m/s^2	9 m/s^2	10 m/s^2
0.8 to <1.0 m/s	5 m/s^2	6 m/s^2	7 m/s^2	8 m/s^2	9 m/s^2
<0.8 m/s	4 m/s^2	5 m/s^2	6 m/s^2	7 m/s^2	8 m/s^2

Tailoring

The PBT protocol was divided into 11 blocks of two to four perturbations and, after each block, the participants were asked to rate their perceived difficulty and anxiety on a scale from 1 to 5 (see Table SM2.2). The perturbation intensity was increased following the block if all of the following criteria were met: 1) the combined rating of perceived anxiety and difficulty was four or less, 2) the participant avoided falling in all four perturbations in the previous block, and 3) the participant felt comfortable increasing the intensity. If any of these criteria were not met, the perturbation intensity remained at the highest tolerated level.

eAppendix 1, Table 2 – The scales on which the participants rated the perceived difficulty and anxiety after each block of four perturbations.

**A. Anxiety - On a scale from 1 to 5, how anxious are you now?**				
**1**	**2**	**3**	**4**	**5**
Not at all anxious	Just a little anxious	Mildly anxious	Moderately anxious	Extremely anxious
**B. Difficulty - On a scale from 1 to 5, how difficult were the last trials?**				
**1**	**2**	**3**	**4**	**5**
Not at all difficult	Somewhat difficult	Difficult	Very difficult	Too difficult

Who provided?

A trained research staff member with a sports science background (JEN) conducted all training sessions in both groups.

How?

A trained research staff member supervised all training sessions on a one-to-one basis.

Where?

All training sessions were conducted in the same laboratory at the Department of Health, Technology, and Science, Aalborg University (Fredrik Bajers Vej 7A2-107, Aalborg, DK-9000, Denmark).

© 2023 Nørgaard JE et al. *JAMA Network Open.*

How well?

**eAppendix 1, Table 3 –** Adherence to the perturbation-based balance training intervention		
Adherence, No. completed sessions	253/280 sessions	90% of all sessions were completed
No. completing entire intervention	53/70	76% completed the entire training intervention
No. completing at least 75% of the intervention	63/70	90% completed at least 75% of the training intervention
No. completing less than 75% of the intervention	7/70	10% completed less than 75% of the training intervention

.

eAppendix 1, Table 4 – Summary of reasons for missed practice in the perturbation-based balance training group

Reason	No. of missed practices	Percentage of all sessions
Injury not obtained during training	10	4%
Injury obtained during training	2	1%
Personal reasons*	7	3%
Excessive anxiety during training	5	2%
Excessive tiredness during training	3	1%

* Personal reasons included logistical issues, lack of motivation, and changes in personal relations forcing a stoppage in participation

eAppendix 1, Table 5 – Maximal intensity reached during each perturbation-based balance training session

Perturbation intensity level	Session 1 slip only	Session 2 trip only	Session 3 mixed	Session 4 mixed
Level 1, n (%)	32 (51)	8 (13)	32 (54)	26 (50)
Level 2, n (%)	10 (16)	14 (23)	6 (10)	7 (14)
Level 3, n (%)	6 (10)	9 (15)	5 (8)	5 (10)
Level 4, n (%)	1 (2)	4 (6)	2 (3)	7 (14)
Level 5, n (%)	14 (22)	27 (44)	14 (24)	6 (12)

eAppendix 1, Table 6 – Average perceived anxiety reached for each block during the perturbation-based balance training sessions. Rated on a scale on 1-5 (higher scores indicate more anxiety).

	Block 1	Block 2	Block 3	Block 4	Block 5	Block 6	Block 7	Block 8	Block 9	Block 10
Session 1* Mean (SD)	1.99 (0.94)	1.90 (0.92)	1.76 (0.93)	1.63 (0.85)	1.60 (0.83)	1.56 (0.70)	1.54 (0.71)	1.48 (0.71)	1.47 (0.75)	1.34 (0.62)
Session 2♦	1.42 (0.72)	1.28 (0.62)	1.25 (0.56)	1.27 (0.57)	1.30 (0.58)	1.26 (0.57)	1.23 (0.55)	1.22 (0.54)	1.20 (0.53)	1.18 (0.61)

© 2023 Nørgaard JE et al. *JAMA Network Open.*

Mean (SD)										
Session 3♣ Mean (SD)	1.75 (0.97)	1.62 (0.82)	1.55 (0.83)	1.54 (0.79)	1.53 (0.79)	1.53 (0.77)	1.50 (0.75)	1.47 (0.70)	1.45 (0.72)	1.31 (0.67)
Session 4♣ Mean (SD)	1.71 (0.88)	1.53 (0.66)	1.58 (0.66)	1.57 (0.63)	1.54 (0.61)	1.57 (0.64)	1.47 (0.58)	1.49 (0.61)	1.47 (0.61)	1.32 (0.55)

* slip only session; ♦ trip only session; ♣ mixed slip and trip session

eAppendix 1, Table 7 – Average perceived difficulty rated for each block during the perturbation-based balance training sessions. Rated on a scale of 1-5 (higher scores indicate greater difficulty).

	Block 1	Block 2	Block 3	Block 4	Block 5	Block 6	Block 7	Block 8	Block 9	Block 10
Session 1* Mean (SD)	2.65 (0.79)	2.56 (0.74)	2.46 (0.78)	2.34 (0.79)	2.32 (0.87)	2.32 (0.77)	2.20 (0.82)	2.14 (0.80)	2.05 (0.77)	1.61 (0.68)
Session 2♦ Mean (SD)	1.67 (0.79)	1.55 (0.76)	1.61 (0.70)	1.66 (0.75)	1.81 (0.91)	1.85 (0.89)	1.80 (0.90)	1.78 (0.92)	1.70 (0.80)	1.20 (0.54)
Session 3♣ Mean (SD)	1.90 (0.78)	1.82 (0.72)	1.85 (0.73)	2.03 (0.74)	2.05 (0.89)	1.97 (0.76)	2.08 (0.82)	1.92 (0.81)	1.97 (0.88)	1.59 (0.66)
Session 4♣ Mean (SD)	2.07 (0.81)	1.91 (0.75)	2.00 (0.72)	2.00 (0.73)	2.07 (0.77)	2.12 (0.78)	2.00 (0.78)	1.96 (0.76)	1.92 (0.76)	1.55 (0.70)

* slip only session; ♦ trip only session; ♣ mixed slip and trip session

eAppendix 1, Table 8 – Adherence to the treadmill walking intervention

Adherence, completed sessions, median [IQR]	259/280 sessions	93% completed of the session were completed
No. completing the entire intervention,	53/70	76% completed the entire training intervention
No. completing at least 75% of the intervention	68/70	97% completed at least 75% of the training intervention
No. completing less than 75% of the intervention	2/70	3% completed less than 75% of the training intervention

eAppendix 1, Table 9 – Summary of reasons for missed practice in the treadmill walking group

Reason	No. of missed practices	Percentage of all sessions
Injury not obtained during training	11	4%
Personal reasons*	10	4%

* Personal reasons included logistical issues, lack of motivation, and changes in personal relations forcing stoppage in participation

© 2023 Nørgaard JE et al. *JAMA Network Open.*

eReferences

1. Pai YC, Bhatt TS. Repeated-Slip Training: An Emerging Paradigm for Prevention of Slip-Related Falls Among Older Adults. Phys Ther. 2007;87(11):1478-1491. doi:10.2522/ptj.20060326

2. Mansfield A, Wong JS, Bryce J, Knorr S, Patterson KK. Review and Meta-Analysis of. 2015;95(5).

3. Grabiner MD, Crenshaw JR, Hurt CP, Rosenblatt NJ, Troy KL. Exercise-based fall prevention: Can you be a bit more specific? Exerc Sport Sci Rev. 2014;42(4):161-168. doi:10.1249/JES.0000000000000023

4. Berg WP, Alessio HM, Mills EM, Tong C. Circumstances and consequences of falls in independent community-dwelling older adults. Age Ageing. 1997;26(4):261-268. doi:10.1093/ageing/26.4.261

5. Luukinen H, Herala M, Koski K, et al. Fracture risk associated with a fall according to type of fall among the elderly. Osteoporos Int. 2000;11(7):631-634. doi:10.1007/s001980070086

6. Karamanidis K, Epro G, McCrum C, König M. Improving Trip- A nd Slip-Resisting Skills in Older People: Perturbation Dose Matters. Exerc Sport Sci Rev. 2020;48(1):40-47. doi:10.1249/JES.0000000000000210

7. Okubo Y, Sturnieks DL, Brodie MA, Duran L, Lord SR, Newman A. Effect of Reactive Balance Training Involving Repeated Slips and Trips on Balance Recovery among Older Adults: A Blinded Randomized Controlled Trial. Journals Gerontol - Ser A Biol Sci Med Sci. 2019;74(9):1489-1496. doi:10.1093/gerona/glz021

8. Brodie MA, Okubo Y, Sturnieks DL, Lord SR. Optimizing successful balance recovery from unexpected trips and slips. J Biomech Sci Eng. 2018;13(4):1-10. doi:10.1299/jbse.17-00558

9. Dijkstra BW, Horak FB, Kamsma YPTT, Peterson DS. Older adults can improve compensatory stepping with repeated postural perturbations. Front Aging Neurosci. 2015;7(OCT):1-8. doi:10.3389/fnagi.2015.00201

10. Epro G, Mierau A, Mc Crum C, Leyendecker M, Brüggemann GP, Karamanidis K. Retention of gait stability improvements over 1.5 years in older adults: Effects of perturbation exposure and triceps surae neuromuscular exercise. J Neurophysiol. 2018;119(6):2229-2240. doi:10.1152/jn.00513.2017

11. König M, Epro G, Seeley J, Catalá-lehnen P, Potthast W, Karamanidis K. Retention of improvement in gait stability over 14 weeks due to trip-perturbation training is dependent on perturbation dose. J Biomech. 2019;84:243-246. doi:10.1016/j.jbiomech.2018.12.011

12. Allin LJ, Brolinson PG, Beach BM, et al. Perturbation-based balance training targeting both slip- And trip-induced falls among older adults: A randomized controlled trial. BMC Geriatr. 2020;20(1):1-13. doi:10.1186/s12877-020-01605-9

13. Wang Y, Bhatt T, Liu X, et al. Can treadmill-slip perturbation training reduce immediate risk of over-ground-slip induced fall among community-dwelling older adults? J Biomech. 2019;84:58-66. doi:10.1016/j.jbiomech.2018.12.017

14. Epro G, McCrum C, Mierau A, Leyendecker M, Brüggemann GP, Karamanidis K. Effects of triceps surae muscle strength and tendon stiffness on the reactive dynamic stability and adaptability of older female adults during perturbed walking. J Appl Physiol. 2018;124(6):1541-1549. doi:10.1152/japplphysiol.00545.2017

15. Okubo Y, Schoene D, Lord SR. Step training improves reaction time, gait and balance and reduces falls in older people: A systematic review and meta-analysis. Br J Sports Med. 2017;51(7):586-593. doi:10.1136/bjsports-2015-095452

© 2023 Nørgaard JE et al. *JAMA Network Open.*

16. Mansfield A, Wong JS, Bryce J, Knorr S, Patterson KK. Does Perturbation-Based Balance Training Prevent Falls? Systematic Review and Meta-Analysis of Preliminary Randomized Controlled Trials. Phys Ther. 2015;95(5):700-709. doi:10.2522/ptj.20140090

17. Mccrum C, Karamanidis K, Meijer K. Older adults demonstrate interlimb transfer of reactive gait adaptations to repeated unpredictable gait perturbations. Published online 2020:39-49.

18. Tanvi B, Feng Y, Yi-Chung P. Learning to resist gait-slip falls: Long-term retention in community-dwelling older adults. Arch Phys Med Rehabil. 2012;93(4):557-564. doi:10.1016/j.apmr.2011.10.027

19. Kang HG, Dingwell JB. Separating the effects of age and walking speed on gait variability. Gait Posture. 2008;27(4):572-577. doi:10.1016/j.gaitpost.2007.07.009

20. Nørgaard JE, Andersen S, Ryg J, et al. Effects of treadmill slip and trip perturbation-based balance training on falls in community-dwelling older adults (STABILITY): Study protocol for a randomised controlled trial. BMJ Open. 2022;12(2):1-10. doi:10.1136/bmjopen-2021-052492

21. Lee A, Bhatt T, Liu X, Wang Y, Pai Y chung C. Can higher training practice dosage with treadmill slip-perturbation necessarily reduce risk of falls following overground slip? Gait Posture. 2018;61(January 2017):387-392. doi:10.1016/j.gaitpost.2018.01.037

22. Lee A, Bhatt T, Liu X, Wang Y, Wang S, Pai YC (Clive). Can Treadmill Slip-Perturbation Training Reduce Longer-Term Fall Risk Upon Overground Slip Exposure? J Appl Biomech. Published online 2020:1-9. doi:10.1123/jab.2019-0211

23. Takazono PS, Ribeiro de Souza C, Ávila de Oliveira J, Coelho DB, Teixeira LA. High contextual interference in perturbation-based balance training leads to persistent and generalizable stability gains of compensatory limb movements. Exp Brain Res. 2020;238(5):1249-1263. doi:10.1007/s00221-020-05806-x

24. Harper SA, Beethe AZ, Dakin CJ, Bolton DAE. Promoting generalized learning in balance recovery interventions. Brain Sci. 2021;11(3):NA. doi:10.3390/brainsci11030402

25. Wang Y, Wang S, Lee A, Pai YC, Bhatt T. Treadmill-gait slip training in community-dwelling older adults: mechanisms of immediate adaptation for a progressive ascending-mixed-intensity protocol. Exp Brain Res. 2019;237(9):2305-2317. doi:10.1007/s00221-019-05582-3

26. Witham MD, Stott DJ. Conducting and reporting trials for older people. Age Ageing. 2017;46(6):889-894. doi:10.1093/ageing/afx153

27. Lee A, Bhatt T, Liu X, Wang Y, Wang S, Pai Y chung CCC. Can treadmill slip-perturbation training reduce longer-term fall risk upon overground slip exposure? J Appl Biomech. 2020;36(5):298-306. doi:10.1123/JAB.2019-0211

© 2023 Nørgaard JE et al. *JAMA Network Open.*

eFigure. Plot of the Cox Survival Analysis

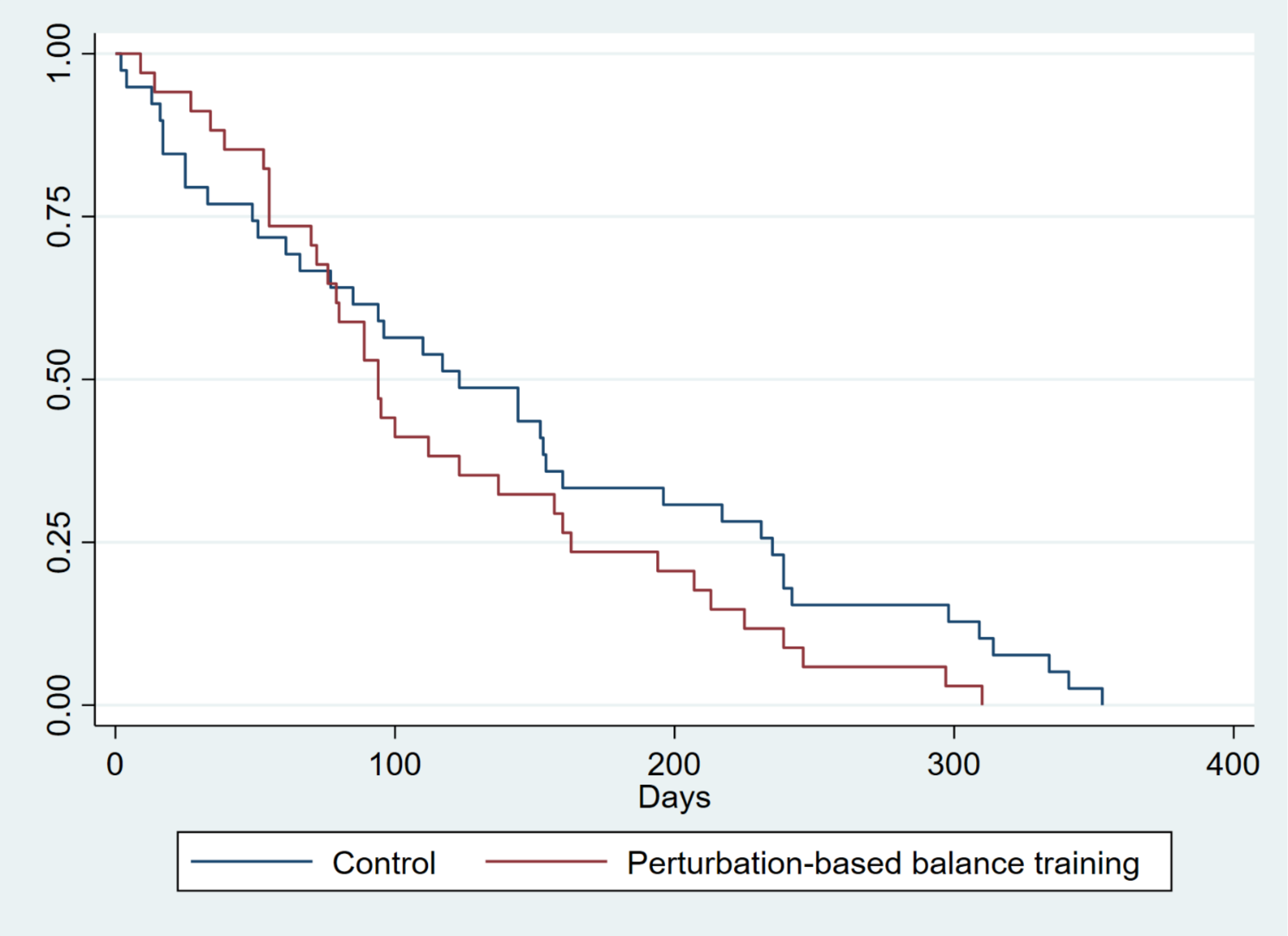

© 2023 Nørgaard JE et al. *JAMA Network Open.*

eAppendix 2. STATA Codes Used in the Primary Analyses

STATA code for count outcomes

This code was used for the outcomes; daily life fall rate, daily life fall-related injury rate, daily life fall-related healthcare contact rate, daily life slip-related fall rate, and daily life trip-related fall rate. All outcomes were found to be overdispersed:

bnreg [insert variable] i.intervention, irr exposure(personyear)

Model adjusting for age, sex, and previous falls:

bnreg [insert variable] i.intervention age i.sex i.prev_faller, irr exposure(personyear)

STATA code for binary outcomes

This code was used for the outcomes; participants with atleast one daily life fall, participants with at least one daily life fall-related injury, and participants with at least one daily life fall-related healthcare contact.

glm [insert variable] i.intervention, fam(poisson) link(log) vce(robust) eform

Model adjusting for age, sex, and previous falls:

glm [insert variable] i.intervention age i.sex i.prev_faller, fam(poisson) link(log) vce(robust) eform

STATA code for time to first fall

stset firstfall, failure(prop_faller == 1)

stcox intervention

estat phtest

Model adjusting for age, sex, and previous falls:

stset firstfall, failure(prop_faller == 1)

stcox intervention age i.sex i.prev_faller

estat phtest

© 2023 Nørgaard JE et al. *JAMA Network Open.*

eTable 1. Reasons for Missing Laboratory Data in Each Group

Reasons	PBT group (n=70)<br>Missing data from 19 (27%)	Control group (n=70)<br>Missing data from 25 (36%)
Injury/illness, not related to training<br>No. participants (%)	11 (16%)	14 (20%)
Personal reasons<br>No. participants (%)	6 (9%)	11 (14%)
Anxiety during training<br>No. participants (%)	2 (3%)	-

© 2023 Nørgaard JE et al. *JAMA Network Open.*

eTable 2. Between-Group Comparison of Participants With Missing Laboratory Fall Data

Variable	With missing data in PBT group (n=19)	With missing data in CONTROL group (n=25)	P-value
Age (years), mean (SD)	73 (5)	72 (5)	0.72 $^{\alpha}$
Sex, no. female (%)	14 (74)	14 (56)	0.34 $^{\gamma}$
Frailty*, median (IQR)	3 (1-3)	2 (1-3)	0.38 $^{\beta}$
Function of daily activities♦, median (IQR)	2 (2-3)	2 (1-3)	0.27 $^{\beta}$
Medication, median (IQR)	4 (1-7)	3 (0-4)	0.13 $^{\beta}$
Previous fallers, no. fallen (%)	9 (32)	8 (47)	0.36 $^{\gamma}$
Baseline laboratory falls, Rate (95% CI)	0.95 (0.67-1.23)	0.96 (0.71-1.21)	0.95 $^{\Sigma}$
Cognition♣, median (IQR)	26 (24-26)	26 (24-28)	0.30 $^{\beta}$
Physical function•, median (IQR)	11 (11-12)	12 (11-12)	0.88 $^{\beta}$
Executive function©, median (IQR)	54.62 (37.16-63.52)	44.65 (28.83-63.52)	0.47 $^{\beta}$

* Tilburg Frailty indicator (score 0-15; lower is better); ♦ The Vulnerable Elderly-13 Survey (score 0-10; lower is better); ♣ The Short Orientation-Memory-Concentration Test (score 0-28; higher is better); • The Short Physical Performance Battery (0-12; higher is better); © Trail Making Test Part A subtracted from Part B (lower score implied better performance), $^{\alpha}$ Unpaired sample t-test; $^{\beta}$ Wilcoxon signed rank test; $^{\gamma}$ Fisher's exact test; $^{\Sigma}$ Poisson regression with bootstrapping; bold text indicates significant between group differences

© 2023 Nørgaard JE et al. *JAMA Network Open.*

eTable 3. Laboratory Fall Rate Results

Table SM4.2 – Results for the laboratory fall rates

	PBT Rate (95% CI)	Control Rate (95% CI)	Between-group IRR (95% CI)
Pre-training	0.69 (0.49 to 0.88)	0.73 (0.53 to 0.93)	0.94 (0.63 to 1.40)
Post-training	0.14 (0.05 to 0.23)	0.69 (0.48 to 0.89)	0.20 (0.10 to 0.41)
6-month follow-up	0.28 (0.14 to 0.42)	0.59 (0.39 to 0.80)	0.47 (0.26 to 0.86)
12-month follow-up	0.23 (0.10 to 0.35)	0.62 (0.39 to 0.84)	0.37 (0.19 to 0.72)

© 2023 Nørgaard JE et al. *JAMA Network Open.*

eAppendix 3. Results of Poisson's Regression With Bootstrapping

The statistical analysis plan stated that count outcomes would be evaluated using Poisson's regression with bootstrapping. However, when analysing the model fits after data collection, it was concluded that a negative binomial regression was more appropriate and applied (Negative Binomial Regression: AIC = 390.2, BIC = 399.0; Poisson's with Bootstrapping: AIC = 445.4, BIC = 451.3). The analysis results using the preregistered method are reported below to accommodate the suspicion of study misconduct. All analyses are conducted in STATA using the following code:

poisson [insert variable] i.intervention, irr exposure(personyear)

	PBT group (n = 70)	Control group (n = 70)	Absolute difference	Relative difference
**Person years of follow-up**	71.5	70.4	-	-
**No. of falls (rate, 95% CI)**	62<br>(0.89, 0.53 to 1.25)	78<br>(1.14, 0.68 to 1.59)	-0.23<br>[-0.79 to 0.32]	0.78<br>[0.44 to 1.38]
**No. of fall-related injuries (rate, 95% CI)**	25<br>(0.36, 0.18 to 0.54)	34<br>(0.50, 0.28 to 0.71)	-0.14<br>[-0.41 to 0.14]	0.72<br>[0.37 to 1.40]
**No. of fall-related healthcare contacts (rate, 95% CI)**	2<br>(0.03, -0.01 to 0.07)	8<br>(0.12, 0.04 to 0.20)	-0.09<br>[-0.18 to 0.00]	0.25<br>[0.05 to 1.16]
**No. of daily life slip falls (rate, 95% CI)**	9<br>(0.13, 0.03 to 0.23)	13<br>(0.19, 0.03 to 0.35)	-0.06<br>[-0.28 to 0.16]	0.68<br>[0.21 to 2.16]
**No. of daily life trip falls (rate, 95% CI)**	27<br>(0.39, 0.10 to 0.67)	33<br>(0.48, 0.24 to 0.72)	-0.09<br>[-0.47 to 0.28]	0.81<br>[0.33 to 1.94]

© 2023 Nørgaard JE et al. *JAMA Network Open.*

eTable 4. Results of Sensitivity Analysis Adjusting for Age, Sex, and Previous Falls

Outcome	PBT group (n = 70)	Control group (n = 70)	Absolute Difference	Relative difference
**Person years of follow-up**	71.5	70.4	-	-
**No. of falls (rate, 95% CI)[ab]**	62 (0.86, 0.58 to 1.14)	78 (1.16, 0.80 to 1.52)	-0.30 [-0.75 to 0.15]	0.74 [0.48 to 1.16]
**Participants with at least one fall (%)**	34 (49%)	39 (57%)	-0.07 [-0.22 to 0.08]	0.88 [0.65 to 1.17]
**Participants with two or more falls (%)**	11 (16%)	15 (21%)	-0.05 [-0.17 to 0.06]	0.75 [0.39 to 1.43]
**Time to first fall, days (median, IQR)**	94 (91 to 148)	142 (107 to 177)	-	1.44 [0.88 to 2.36]
**No. of fall-related injuries (rate, 95% CI)[b]**	25 (0.35, 0.19 to 0.50)	34 (0.48, 0.29 to 0.67)	-0.14 [-0.38 to 0.11]	0.72 [0.40 to 1.30]
**Participants with at least one fall-related injury (%)**	17 (24%)	24 (34%)	-0.10 [-0.25 to 0.05]	0.71 [0.43 to 1.19]
**No. of fall-related healthcare contact (rate, 95% CI)[b]**	2 (0.03, -0.01 to 0.07)	8 (0.12, 0.04 to 0.20)	-0.09 [-0.18 to 0.00]	0.25 [0.06 to 1.25]
**Participants with at least one fall-related hospital contact (%)**	2 (3%)	7 (10%)	-0.07 [-0.15 to 0.01]	0.29 [0.06 to 1.33]
**No. of slip falls (rate, 95% CI)[b]**	9 (0.13, 0.03 to 0.23)	13 (0.18, 0.07 to 0.30)	-0.05 [-0.21 to 0.10]	0.71 [0.27 to 1.90]
**No. of trip falls (rate, 95% CI)[b]**	26 (0.36, 0.19 to 0.53)	33 (0.50, 0.27 to 0.74)	-0.08 [-0.47 to 0.31]	0.71 [0.36 to 1.40]

[a] primary outcome; [b] over-dispersed data, negative binomial regression; PBT = Perturbation-based balance training

eTable 5. Results of Sensitivity Analysis Following the Per-Protocol Principle

Outcome	PBT group (n = 63)	Control group (n = 68)	Absolute Difference	Relative difference
**Person years of follow-up**	65.3	69.3	-	-
**No. of falls (rate, 95% CI)[ab]**	60 (0.95, 0.61 to 1.29)	78 (1.16, 0.78 to 1.54)	-0.21 [-0.72 to 0.30]	0.82 [0.50 to 1.33]
**Participants with at least one fall (%)**	33 (47%)	39 (57%)	-0.05 [-0.22 to 0.12]	0.91 [0.67 to 1.25]
**Participants with two or more falls (%)**	10 (14%)	15 (21%)	-0.05 [-0.22 to 0.12]	0.72 [0.35 to 1.49]
**Time to first fall, days (median, IQR)**	119 (90 to 148)	142 (107 to 177)	-	1.41 [0.87 to 2.28]
**No. of fall-related injuries (rate, 95% CI)[b]**	23 (0.36, 0.19 to 0.53)	33 (0.49, 0.29 to 0.69)	-0.13 [-0.39 to 0.14]	0.74 [0.40 to 1.39]
**Participants with at least one fall-related injury (%)**	16 (25%)	24 (35%)	-0.10 [-0.26 to 0.06]	0.72 [0.42 to 1.23]
**No. of fall-related healthcare contacts (rate, 95% CI)[b]**	2 (0.03, -0.01 to 0.08)	8 (0.12, 0.03 to 0.21)	-0.09 [-0.19 to 0.01]	0.27 [0.05 to 1.31]
**Participants with at least one fall-related hospital contact (%)**	2 (3%)	7 (10%)	-0.07 [-0.16 to 0.01]	0.31 [0.07 to 1.44]
**No. of slip falls (rate, 95% CI)[b]**	9 (0.13, 0.03 to 0.25)	13 (0.19, 0.06 to 0.32)	-0.05 [-0.22 to 0.12]	0.73 [0.27 to 2.03]
**No. of trip falls (rate, 95% CI)[b]**	26 (0.41, 0.19 to 0.63)	33 (0.49, 0.25 to 0.73)	-0.08 [-0.41 to 0.24]	0.84 [0.41 to 1.72]

[a] primary outcome; [b] over-dispersed data, negative binomial regression; PBT = Perturbation-based balance training

© 2023 Nørgaard JE et al. *JAMA Network Open.*

eTable 6. Results of Sensitivity Analysis Stratifying Participants by Fall History

A – With a history of falls within the previous 12 months

	PBT group (n = 28)	Control group (n = 29)	Absolute difference	Relative difference
**Person years**	28.9	28.9	-	-
**No. of falls (rate)**[a]	48 (1.67, 0.96 to 2.37)	53 (1.83, 1.07 to 2.59)	-0.16 [-1.20 to 0.87]	0.91 [0.50 to 1.65]

B - Without a history of falls within the previous 12 months

	PBT group (n = 42)	Control group (n = 41)	Absolute difference	Relative difference
**Person years**	42.5	41.4	-	-
**No. of falls (rate)** [a]	14 (0.33, 0.17 to 0.48)	25 (0.60, 0.31 to 0.90)	-0.27 [-0.60 to 0.05]	0.55 [0.28 to 1.08]

[a] over-dispersed data, negative binomial regression

© 2023 Nørgaard JE et al. *JAMA Network Open.*

eAppendix 4. Supplemental Methods and Results of Multiple Imputations

Due to a high number of missing data in the laboratory fall outcome, we conducted a sensitivity analysis with multiple imputations. A multivariant imputation by chained equations model imputes missing data across all time points and participants, and this model is appropriate when data are missing at random. The imputed data were applied in the analytic model as the non-imputed data using the “mi estimate” (Poisson regression for laboratory fall rates; linear mixed model for the proportion of slip and trip fallers). All imputations were conducted in STATA version 17.0 (Statacorp LLC, College Station, Texas, USA). The variables used in the model are outlined in the table below:

Variables without missing data are used for the prediction of imputed values on other variables	Variables with missing data that were imputed and used for the prediction of imputed values on other variables
1. Baseline laboratory trips<br>2. Baseline laboratory slips<br>3. Baseline Age<br>4. Baseline Sex<br>5. Baseline previous daily life falls<br>6. Baseline Frailty<br>7. Baseline Physical activity levels<br>8. Baseline Activity of daily living functionality<br>9. Baseline No. of medications<br>10. Baseline Gait speed<br>11. Baseline Dual-task Gait speed<br>12. Baseline Stepping reaction speed<br>13. Baseline Static balance*<br>14. Baseline Dual task Static balance*<br>15. Baseline Short Physical Performance Battery<br>16. Baseline Executive function<br>17. Baseline Fear of Falling<br>18. Baseline Quality of life	1. Post-training laboratory trips<br>2. 6-month follow-up laboratory trips<br>3. 12-month follow-up laboratory trips<br>4. Post-training laboratory slips<br>5. 6-month laboratory slips<br>6. 12-month follow-up laboratory slips

* CoM displacement speed

A two-stage approach was employed for each of the imputed outcomes. First, a pilot analysis using 20 imputations was conducted. Second, using these results, the actual number of imputations was calculated using the “how_many_imputations” command.[1]

Results of analysis

The rate of laboratory falls in the analysis following multiple imputations was similar to the primary analysis. A significantly lower rate of laboratory falls was present in the PBT compared to the control group at the post-

© 2023 Nørgaard JE et al. *JAMA Network Open.*

training test (IRR: 0.21, 95% CI, 0.10 to 0.44, 12 imputations) 6-month follow-up (IRR: IRR: 0.52, 95% CI, 0.29 to 0.91, 16 imputations), and 12-month follow-up (IRR: 0.44, 95% CI, 0.24 to 0.80, 27 imputations).

© 2023 Nørgaard JE et al. *JAMA Network Open.*

eTable 7. Adverse Events Reported During the Study

Date	Description
April 25th 2021	What: Tiredness<br>During: TW session 2
May 6th 2021	What: Muscle sprain in quadriceps<br>During: PBT session 1
May 17th 2021	What: Sciatic nerve irritation<br>During: following PBT sessions 1 and 2<br>Note: The participant has a history of sciatic nerve irritations
July 2nd 2021	What: Knee injury (clinical suspicion of ligament injury)<br>During: PBT session 3
July 27th 2021	What: Foot pain<br>When: TW session 2<br>Note: Participant has a Charcot's foot
August 2nd 2021	What: Mental tiredness<br>During: PBT session 1
September 21th 2021	What: Delayed Onset Muscle Soreness in the calf<br>During: following PBT sessions 1 and 2
September 23rd 2021	What: Hamstring soreness<br>During: PBT session 1
September 27th 2021	What: Groin discomfort<br>During: PBT session 3
September 28th 2021	What: Hip soreness<br>During: PBT sessions 1 and 2<br>Note: participant informs that hip soreness is usual during physical exercise
September 28th 2021	What: Hamstring soreness<br>During: PBT session 1
September 29th 2021	What: Knee soreness<br>During: PBT session 2<br>Note: participant has knee arthritis
October 1th 2021	What: Mental tiredness<br>During: PBT session 2
October 5th 2021	What: Hip soreness<br>During: PBT session 3
October 7th 2021	What: Mental tiredness<br>During: PBT session 2
October 13th 2021	What: Knee soreness<br>During: PBT session 2
November 18rd 2021	What: Pain in the foot<br>When: PBT session 1<br>Note: Participant informs that foot pain is often experienced after physical exercise
November 25th 2021	What: Pain in the ankle<br>During: PBT session 4<br>Note: The participant has previously broken the ankle and often experiences pain during physical exercise
April 7th 2022	What: Knee soreness<br>During: PBT session 2<br>Note: participant has knee arthritis
April 28th 2022	What: Hip soreness<br>During: PBT session 3

© 2023 Nørgaard JE et al. *JAMA Network Open.*

	Note: Participant informs that hip pain is often experienced after physical exercise

© 2023 Nørgaard JE et al. *JAMA Network Open.*
