# Supplementary material for: Effect of Treadmill Perturbation-Based Balance Training on Fall Rates in Community-Dwelling Older Adults: A Randomized Clinical Trial
Source: JAMA Netw Open. 2023 Apr 20;6(4):e238422. doi: 10.1001/jamanetworkopen.2023.8422 (PMC10119738; doi:10.1001/jamanetworkopen.2023.8422)
Supplement: Supplement 3. — Data Sharing Statement [file jamanetwopen-e238422-s003.pdf]

Data Sharing Statement

Nørgaard. Effect of Treadmill Perturbation-Based Balance Training on Fall Rates in Community-Dwelling Older Adults. *JAMA Netw Open*. Published April 20, 2023. doi:10.1001/jamanetworkopen.2023.8422

Data
Data available: Yes
Data types: Deidentified participant data
How to access data: Email: Jens.noergaard@rn.dk
When available: With publication

Supporting Documents
Document types: None

Additional Information
Who can access the data: Anyone requesting the data
Types of analyses: For any purpose
Mechanisms of data availability: With investigator support
